# Supplementary material for: Learning the fitness dynamics of pathogens from phylogenies
Source: Nature. 2025 Jan 1;637(8046):683–90. doi: 10.1038/s41586-024-08309-9 (PMC11735385; doi:10.1038/s41586-024-08309-9)
Supplement: Supplementary file 1 — This file contains Supplementary Information sections 1–4, Supplementary Figs. 1–19, Supplementary Tables 1–5 and references. [file 41586_2024_8309_MOESM1_ESM.pdf]

nature portfolio

https://doi.org/10.1038/s41586-024-08309-9

Supplementary information

Learning the fitness dynamics of pathogens from phylogenies

In the format provided by the authors and unedited

Supplementary information

Manuscript: Learning the fitness dynamics of pathogens from phylogenies

Authors:
Noémie Lefrancq[1,2,3,*], Loréna Duret[1], Valérie Bouchez[3,4], Sylvain Brisse[3,4], Julian Parkhill[2,+], Henrik Salje[1,+]

Affiliations:
1. Department of Genetics, University of Cambridge, Cambridge, UK
2. Department of Veterinary Medicine, University of Cambridge, Cambridge, UK
3. Department of Biosystems Science and Engineering, ETH Zürich, 4009, Basel, Switzerland
4. Institut Pasteur, Université de Paris, Biodiversity and Epidemiology of Bacterial Pathogens, Paris, France
5. National Reference Center for Whooping Cough and Other Bordetella Infections, Paris, France

+ These authors jointly supervised this work
* Corresponding author: ncmjl2@cam.ac.uk

Supplementary Text

Supplementary Text 1: Theoretical index behaviour

We provide here the analytical computation of the index behaviour in different populations. Let's recall Equation 1: we define the *Index* of each isolate i in its population at time t as:

$$Index(i) = \sum_{d=0}^{\infty} D_i(d,t) \cdot b^d$$

[Eq. 1]

With $D_i(d,t)$ the distance distribution - in number of mutations or evolutionary time (branch length) - from the isolate i to the rest of the population (internal and terminal nodes) at that time t (Fig. 1) and b^d, the kernel setting the weight of each distance d. b is the bandwidth, $b \in]0,1[$, which is a parameter to set, linked to the timescale (Table S1).

Using the probability $P_c(s = \frac{d}{\mu l}, t)$, for any pair of sequences sampled at time t, to coalesce some time $s = \frac{d}{\mu l}$ in the past, with μ being the rate at which the pathogen accumulates mutations per site and per unit of time, and l the length of its genome, we can write:

$$Index(t) = \int_0^{\mu l t} P_c(\frac{u}{\mu l}, t) \cdot b^u \; du$$

[Eq. 2]

Next, we write $P_c(\frac{u}{\mu l}, t)$ for different effective population sizes.

Expected behaviour of the index in a *constant effective population size*

In the simplest case of the structured coalescent process[17], if we consider two individuals from a population of constant size N_e, we can write their probability of coalescing some time s in the past as (Supplementary Fig. 18c):

$$P_c(s = \frac{d}{\mu l}, t) = \frac{1}{K}\frac{1}{N_e} exp(-\frac{s}{N_e}), \qquad if\ s \le t \Leftrightarrow d \le \mu l t$$

$$P_c(s = \frac{d}{\mu l}, t) = 0, \qquad if\ s > t \Leftrightarrow d > \mu l t$$

[Eq. 4]

With K the normalisation constant, so that $\int_0^{\infty} P_c(\frac{u}{\mu l}, t)\, du = 1$.

$$K = \mu l \,(1 - exp(-\frac{t}{\mu l\, N_e}))$$

We can plug Equation 3 in the index definition from Equation 2, making sure we take $s = \frac{d}{\mu l} \Leftrightarrow d = s\mu l$. After simplification it follows that:

$$Index(t) = \frac{(b \cdot exp(-\frac{1}{\mu l N_e}))^{\mu l t} - 1}{(\mu l\, N_e ln(b) - 1)\,(1 - exp(-\frac{t}{N_e}))}, \quad t > 0$$

[Eq. 5]

Which is the behaviour of the index as a function of time, in a constant population size.

Expected behaviour of the index in a *varying population size*

Following the work of Griffiths and Tavaré [18] on the coalescent process in varying population sizes, we can further derive the index in more complex population dynamics. We set the effective population size of our lineage to $\boldsymbol{N_e(t)}$, which can vary through time. We can define the population-size intensity function $\boldsymbol{\Lambda}$ by [1819]:

$$\Lambda_t\ (s)\ =\ \int_0^s \frac{ds'}{N_e(t\ -\ s')},\quad t\ \geq s > 0$$

We assume that $\Lambda(\infty) = \infty$, so that each pair of individuals may be traced back to a common ancestor with probability one [18]. The density λ of Λ is given by [18]:

$$\lambda_t(s)\ = \frac{1}{N_e(t-s)},\quad t\ \geq s > 0$$

It follows that $P_c(s,t)$, i.e. the probability of waiting s time to have the first coalescent event is:

$$P_c(s,t)\ =\ \lambda_t(s)\ exp\ (-\Lambda_t(s)),\quad t\ \geq s > 0$$

We can find back Equation 3, by taking $s = \frac{d}{\mu}$ and plugging in a constant population size $N_e(t)\ =\ N_e$:

$$\Lambda_t(s = \frac{d}{\mu l})\ =\ \int_0^{\frac{d}{\mu l}} \frac{ds'}{N_e} = \frac{d}{\mu l N_e}\ ;\quad \lambda_t(s = \frac{d}{\mu l})\ = \frac{1}{N_e}$$

$$P_c(s = \frac{d}{\mu l}, t)\ = \frac{1}{K}\frac{1}{N_e} exp\ (-\frac{d}{\mu l N_e}),\ \ t\ \geq \frac{d}{\mu l} > 0$$

With K the normalisation constant. Next, we consider the case of exponentially varying population size.

Expected behaviour of the index in an *exponentially growing effective population size.*

We set: $N_e(t)\ =\ N_0 \cdot e^{rt}$, with N_0 the initial population size and r the rate at which the population is growing (Supplementary Fig. 18f). We assume $r > 0$. We can then define the new $\lambda_t(s)$ and $\Lambda_t(s)$:

$$\Lambda_t(s)\ =\ \frac{1}{N_0 r} e^{-rt}(e^{rs}\ -\ 1)$$

And:

$$\lambda_t(s)\ = \frac{1}{N_0} e^{r(s-t)}$$

So that:

$$P_c(s\ = \frac{d}{\mu l}, t)\ =\ \frac{1}{K}\frac{1}{N_0} e^{r(s-t)} exp\ (\frac{1}{N_0 r} e^{-rt}(1 - e^{rs})),\qquad if\ \ t\ \geq s > 0$$

$$P_c(s\ = \frac{d}{\mu l}, t)\ =\ 0,\qquad if\ \ t\ < s$$

[Eq. 6]

With K the normalisation constant so that $\int_0^\infty P_c(\frac{u}{\mu l}, t)\ du\ =\ 1$.

Therefore, we can plug Equation 6 in the index definition from Equation 2, which leads to:

$$Index(t) = \frac{1}{K}\int_0^{\mu l t} \frac{1}{N_0} e^{r(\frac{u}{\mu l}-t)} exp\ (\frac{1}{N_0 r} e^{-rt}(1 - e^{r\frac{u}{\mu l}})) \cdot b^u\ \ du\,,\quad t\ \geq \frac{d}{\mu l} > 0$$

[Eq. 7]

This sum does not have a closed-form expression. However, it can be numerically approximated (Supplementary Fig. 18h).

Expected index behaviour for newly emerging lineage

We can note that in the case of a varying population size (e.g. exponentially varying), the index is dependent on r, the rate at which the population size is varying.

We can derive the index in structured populations that are more complex. For example, we consider here the case of a new lineage expanding in a population (schematic below). Let Pop_A be the ancestral population (schematic below, in grey), with constant effective population size N_A, and Pop_B, an offspring from Pop_A (schematic below, in green), which appeared at time t_B. At time t_B, the effective population size $N_B(t)$ of Pop_B is N_{B_0}. We assume that the Pop_B is growing exponentially ($N_B(t) = N_{B_0} exp(rt)$) through time with rate $r > 1$.

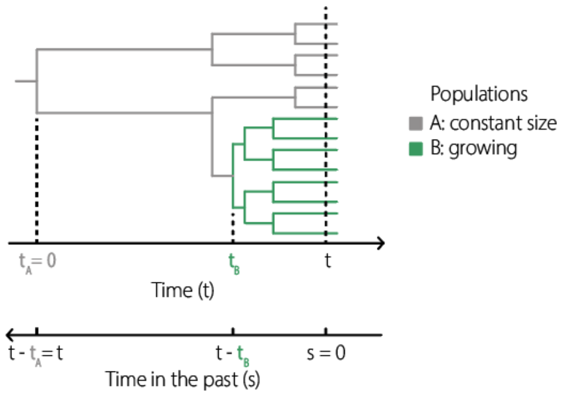

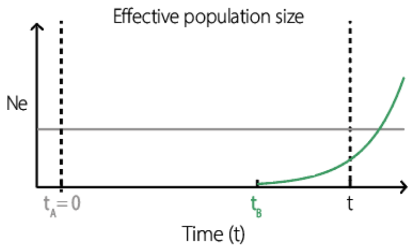

We now write the index of each population. We assume that the appearance of population B has a negligible impact on the index of the individuals sampled from population A. The effective size of population A is constant through time, therefore we can use Equation 5:

$$Index_{Indiv\ in\ Pop\ A}(t) = \frac{\left(b\cdot exp\left(-\frac{1}{\mu l\, N_A}\right)\right)^{\mu l t} - 1}{(\mu l\, N_A ln(b) - 1)\, \left(1 - exp\left(-\frac{t}{N_A}\right)\right)}, \quad t \geq 0$$

Population B is growing exponentially, within population A, therefore writing the index of individuals sampled from this population is more complex. Let's consider an individual sampled from population B. Its probability to coalesce with the rest of the population can be separated in two cases:

- It coalesces with an individual from population B, with probability $P_{c,B\to B}(s,t)$
- Or it coalesces with an individual from population A, with probability $P_{c,B\to A}(s,t)$

The total population through time is: $N_{tot}(t) = N_A + N_B(t)$.

Therefore, the probability of an individual sampled from population B to coalesce with another individual in the population is:

$$P_{c,B\to pop}(s,t) = \frac{N_B(t)}{N_{tot}(t)} P_{c,B\to B}(s,t) + \frac{N_A}{N_{tot}(t)} P_{c,B\to A}(s,t)$$

[Eq. 8]

We can note that $P_{c,BB}(s,t)$ exists only for $t > t_B$ (otherwise population B does not exist yet) and $t - t_B \geq s \geq 0$, and $P_{c,BA}(s,t)$ exists only for $s \geq t - t_B$.

First, let's write $P_{c,B\to B}(s,t)$. As population B is growing exponentially, we can re-use Equation 7:

$$P_{c,B\to B}(s = \frac{d}{\mu l}, t) = \frac{1}{N_{B_0}} e^{r(s-t)} exp\,(\frac{1}{N_{B_0} r} e^{-rt}(1 - e^{rs})), \qquad t \geq t_B \; and \; t - t_B \geq s \geq 0$$

[Eq. 9]

Second, let's write $P_{c,B\to A}(s,t)$. We note that this probability only exists for $s \geq t - t_B$, and the size of population A is constant. So we can rescale this probability:

$$P_{c,B\to A}(s,t) = P_{c,A}(s - t_B, t)$$

We can note that we already wrote this probability earlier in equation 4, so it follows that:

$$P_{c,B\to A}(s,t) = \frac{1}{N_A} exp(-\frac{s}{N_A}), \qquad if \;\; s - t_B > 0$$

[Eq. 10]

We can now plug Equations 9 and 10 into Equation 8, to obtain the index of individuals sampled from population B:

$$Index_{Indiv\ in\ PopB}(t) = \frac{1}{K}(\frac{N_B(t)}{N_{tot}(t)} \int_0^{\mu l(t-t_B)} \frac{1}{N_{B_0}} e^{r(\frac{u}{\mu l}-t)} exp\,(\frac{1}{N_{B_0} r} e^{-r(t-t}(1 - e^{r\frac{u}{\mu l}})) \cdot b^u \, du + \frac{N_A}{N_{tot}(t)} \int_{\mu l(t-t_B)}^{\mu l t} \frac{1}{N_A} exp(-\frac{1}{N_A} \cdot \frac{u}{\mu l}) \cdot b^u \, du\ ) \,, \;\; t \geq t_B > 0$$

[Eq. 11]

With K the normalisation constant so that $\int_0^\infty P_{c,B}(\frac{u}{\mu l}, t)\, du = 1.$

Similarly to Equation 7, this Equation does not have a closed-form expression. However, it can be numerically approximated. Further, we can note that considering only two different populations already makes the index mathematically hard to track, at least without simplifying assumptions.

Supplementary Text 2: Details on simulation study

Simulations to verify the index dynamics

To verify the the expected index dynamics, we simulate trees for different population structures. We use the *sim2.bd.origin* function from the TreeSim package[63]. It simulates trees based on a birth-death model, with set rates of speciation (birth, λ) and extinction (death, μ). A constant effective population size can be simulated by $\lambda = \mu$. An exponentially growing effective population can be simulated by $\lambda > \mu$. To simulate a tree with an emerging lineage, we first simulate separately two trees, one with constant effective population size, and one with an exponentially growing effective population size. Then, we randomly select one tip from the first tree and use this tip as the root of the second tree. In Supplementary Fig. S18, we present those simulations, for three types of effective population sizes: constant, growing, and structured with an emerging lineage. We compare the simulation obtained with the formal expected dynamics (see derivations below). Overall, the simulations verify the formal expected dynamics. Parameters used: time window: 2 years, timescale: 1 year, substitution rate: 4 mutations per year.

We also reproduced sampling bias to check that our formal expected dynamics are correct even in that case. We sampled the sequences generated either taking 10% of the sequences from year 2-8 or only sequences from years 4-6 and 8-10 (and not years 1-3 or 6-8), mimicking common surveillance system biases. In Supplementary Fig. 19, we present those simulations, with 50 replicates each time. Overall, the simulations verify the validity of our approach. Parameters used: time window: 2 years, timescale: 1 year, substitution rate: 4 mutations per year.

Simulation study to test the ability of our approach to detect emerging lineages

To further test our approach to detect emerging lineages, we conducted a large simulation study. We repeatedly simulated timed phylogenetic trees where one lineage expands with a known fitness advantage compared to the background population. We used the package ReMASTER in BEAST2 to perform the simulations using a coalescent-based approach[64]. For each scenario, we simulated a tree by separately simulating the background tree and the emerging tree. Each tree was simulated in a coalescent-based fashion, by setting the effective size of each population. The background effective population size was set to 1000 for 40 time units, after which it decreased given a logistic growth model, reflecting the replacement of the background population by the emerging lineage. The emerging effective population size is set to 50 at T=40 (equivalent to a proportion of 5% in the population), and increases following a logistic growth model. The background tree and the emerging tree are then combined to form one unique tree. Trees are subsequently subsampled to a specific number of sequences. Example of simulated trees are presented in Extended Data Fig. 1.

We employ three different simulation strategies to test our model:

'Full lineage replacement' (Extended Data Fig. 2a-c). We simulate trees until the emerging lineage reaches 95% of the population, which is ensuring the emerging is seen replacing the background population. Each tree contains 1500 tips. We tested six different fitness differences, spanning 0.05/year to 0.5/year. These example values cover a broad range of dynamics ranging from slow to fast lineage replacement, consistent with what we observed in our case study pathogens. We also considered the case of a homogeneous population where no emerging lineage is present. In total, we simulated 700 trees (100 per scenario). In all the scenarios, we find that our method is able to identify

the emerging lineages with near-perfect precision (Extended Data Fig. 2a). *phylowave* is also specific: when no emerging lineage is present, our method does not falsely identify a lineage, in contrast to standard clustering tools, which still define clusters, even in the absence of a lineage with any selective advantage. Further, we quantified the fitness of each lineage by using our logistic model and correctly recovered their fitness (Extended Data Fig. 2b).

'Changing sampling intensity' (Extended Data Fig. 2d). We simulated trees until 10 years post lineage emergence and varied the number of sequences sampled (from 25 to 2500 sequences). We tested 13 different fitness differences, spanning 0.01/year to 10/year. These simulations were done using the same framework as above. For each scenario, we simulated 20 trees. In total, we simulated 3900 trees. Overall we found that the sampling intensity did not impact very much the lineage detection ability. All the lineages with a fitness >1/year were consistently detected, even in trees that were very sparsely sampled.

'Changing sampling window post lineage emergence' (Extended Data Fig. 2e). Lastly, we investigated the impact of the time between lineage emergence and the dates of sequences. As above, we simulated trees with 1500 tips each, however here we varied the time at which the simulation stopped (between 1 and 200 years). For each scenario, we simulated 20 trees. In total, we simulated 4420 trees. We found that lineages with only small fitness advantages require sequences covering longer time periods to be detected. Therefore, a lineage with a fitness advantage as low as 0.02/year, which would take >300 years to become the majority in the population, will not be detected with datasets that do not span that long. However, for larger fitness difference, *phylowave* was able to consistently identify the emerging lineage. We note that the time periods considered can be covered by internal or terminal nodes, not necessarily by sequences (terminal nodes) alone.

Additionally, to investigate how *phylowave* compares to existing approaches, we tested both fastbaps[14] and treestructure[15] on the simulated '*optimal signal*' datasets described above. To test fastbaps, we simulated sequence alignment for each simulated tree using the AliSim tool[65] from IQ-TREE[50]. We simulated an alignment of 3000 positions for each tree, with a substitution rate of 0.001 mutations per site per unit of time, an equal proportion of bases and a JC69 substitution model. We used default parameters to optimise the prior and find the best baps partition. To test treestructure, we ran the *trestruct* function on each simulated phylogeny with a minCladeSize of 30, a significance level of 0.005 and other parameters set to default. For each scenario and for each tree, we then compared the results obtained with each method (Supplementary Fig. 2). An example of the different methods is presented in (Supplementary Fig. 1). We found that our method was consistently better at recovering the population structure (i.e. one emerging lineage and a background population.

Supplementary Text 3: General guidance to use *phylowave* on a dataset.

This general guidance is also available in the README at https://zenodo.org/records/13952222 [Ref: [62]]*, together with the codes.*

phylowave is an approach that summarises changes in population composition in phylogenetic trees of pathogens, allowing for the automatic detection of lineages based on shared fitness and evolutionary relationships. It is currently written in R, with the exception of the fitness model which is written in Stan. In principle, *phylowave* is applicable to any pathogen (e.g. from viruses and bacteria) provided a timed-phylogeny is available and the sampling is representative of the diversity.

Here we describe the general organisation of the pipeline, which is detailed in the following sections:

- Input
- Index computation
- Lineage detection
- Quantification of lineages' fitness
- Lineage-defining mutations

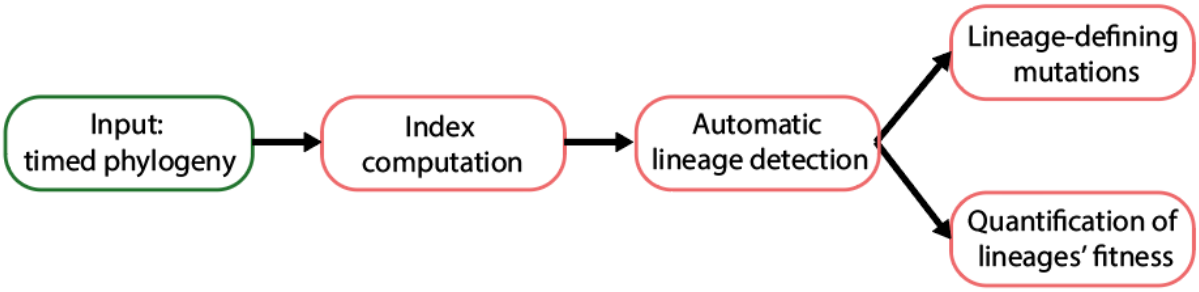

Input

The minimal inputs to use the pipeline are:

- a **time-resolved phylogenetic tree**. The tree must be of class *phylo* and *binary*. The functions *ape::read.tree* and *ape::read.nexus* will enable you to load most trees (of newick or nexus formats, respectively) as objects of class *phylo*. You can test if you tree is binary with the function *ape::is.binary*. If it isn't binary you can always use the function *ape::multi2di* to resolve the polytomies artificially.
- the **sampling times of all tips**. This must be a vector of numerical values, of the same length as the number of tips.

Index computation

To compute the index of all nodes, use the function compute.index, you will need different inputs:

1. Data:
   - the *timed_tree*
   - *metadata* dataframe (see below the details of the *dataset_with_nodes*)
   - *distance matrix*: can be computed from the timed tree with the function *dist.nodes.with.names*

2. Information on the pathogen genome (these are pathogen specific):
    - The genome length of the pathogen considered: *genome_length* (in bp).
    - The mutation rate of the pathogen considered: *mutation_rate* (in bp/genome/year), an average is fine.
3. Index parameters (these are both pathogen-specific and dataset-specific):
    - The *timescale*: timescale (in years), this will be used to compute the bandwidth, see more details below.
    - Window of time on which to search for samples in the population: *wind*, see more details below.

The function outputs a vector containing the index of each node (internal and terminal).

More details on the input of the function:

dataset_with_nodes: To store the data throughout the analysis, we use a main metadata dataframe called dataset_with_nodes which looks like this:

ID	name_seq	time	is.node	Known clade classification	Index
1	name1	t1	'no'		
2	name2	t2	'no'		
3	name3	t3	'no'		
…	…	…	…		
n	namen	tn	'no'		
n+1	n+1	tn+1	'yes'		
…	…	…	…		
2n−1	2n−1	t2n−1	'yes'		

Where n is the number of tips (terminal nodes) in the tree. The column 'Known clade classification' is optional, but is useful to compare the results to existing sequence classifications. The index of each node (internal and terminal) is stored in the column 'Index'.

timescale: The timescale determines the kernel which enables to track lineage emergence dynamically, focusing on short distances between nodes (containing information about recent population dynamics) rather than long distances (containing information about past evolution). The timescale is tailored to the specific pathogen studied and its choice depends on the molecular signal,

as well as the transmission rate. In the study, we used timescales ranging from months (typical of RNA viruses) to years (typical of bacteria). To determine a timescale suitable for your dataset, we recommend thinking about the generation time of the pathogen considered, its mutation rate, and the amount of diversity already accumulated. For example, at the time of the analysis, SARS-CoV-2 was a new pathogen, spreading quickly and accumulating diversity at a rate of ~2 mutations per month. Therefore, a small timescale of less than a year chosen (0.15 years). On the contrary, *Mycobacterium tuberculosis* is an older and relatively slowly spreading pathogen, which accumulates mutations at a rate of ~0.2 mutation per year. A much larger timescale was then chosen (30 years), to reflect this. Ultimately, the best timescale is one that maximises the visualisation of population dynamics. We recommend trying different values.

wind: The choice of wind will depend on the sampling intensity of the dataset. It defines the window of time around each node on which to search for samples in the population. Ultimately it smooths the index dynamics. As a mean of example, for SARS-CoV-2, we set wind to 15 days, as the dataset was intensely sampled. But for *Bordetella pertussis,* which is more sparsely sampled, we chose a wind of 1 year. If wind is too large, then all the nodes are considered to be part of the same time window. If wind is too small, then only the nodes in direct proximity of the node of interest will be considered in the time window, which can result in noisy index dynamics. We recommend choosing a wind value that enables to span multiple sampling times, for example if you have samples and nodes every week, you may choose a wind of ~1-2 months. If you have samples and nodes every year, you may choose a wind of ~2 years.

For an example, see the SARS-CoV-2 code in Supplementary Text 4.

Lineage detection

To run the lineage detection algorithm, use the function *find.groups.by.index.dynamics*, you will need different inputs:

1. Data: *timed_tree* and *metadata* (*dataset_with_nodes*)
2. Lineage detection parameters:
    - *min_descendants_per_tested_node*: to start the analysis, start from nodes that have this minimum number of sequences
    - *min_group_size*: minimum group size, when creating a new potential split
    - *node_support*: numeric value of support of each node (e.g. mutations on the branch leading to the node, or bootstrap support)
    - *threshold_node_support*: threshold on the node support for the nodes to be considered in the detection algorithm
    - *weight_by_time*: size of the window of time on which to compute the weights (NULL or numeric, in years)
    - *weighting_transformation*: type of weighting to use (NULL, inv_freq, inv_sqrt, or inv_log)
    - *max_groups_found*: maximum number of groups to find (Integer)
3. Technical parameters: they do not necessarily need to be updated (see the function documentation for details): *p_value_smooth*, *stepwise_deviance_explained_threshold*,

stepwise_AIC_threshold, *k_smooth*, *parallelize_code*, *number_cores*, *plot_screening*, *keep_track* and *log_y*.

The function outputs multiple elements in a list:

- potential_splits: vector of the nodes included in most complex model tested
- best_dev_explained: vector of the deviance explained by the best models for each number of groups
- first_dev: the null deviance of the initial model (when no lineage is present)
- best_AIC : vector of the AIC of the best models for each number of groups
- best_BIC: vector of the BIC of the best models for each number of groups
- best_summary: list of the summaries of the best models for each number of groups
- best_mod: list of the best models for each number of groups
- best_groups: list of the groups used in the best models for each number of groups
- best_nodes_names: list of the nodes included in the best models for each number of groups

Typically, one chooses a value of *max_groups_found* greater than the expected number of lineages. The algorithm then runs until it finds all those groups, or until it cannot find any significant split anymore. The user can then check the deviance explained by all the models with increasing complexity and choose an adequate number of groups.

Once the split nodes have been defined, the user can then extract the group ID for each node using the function *merge.groups*. One can choose to refine these groups if needed, by setting a minimum number of nodes per group (*group_count_threshold*) or a minimum frequency of each group (*group_freq_threshold*).

For an example, see the SARS-CoV-2 code in Supplementary Text 4.

Post-hoc analyses

Two main post-hoc analyse can be done and are briefly described below.

Quantification of lineages' fitness

To quantify the fitness of each lineage, we developed a multinomial logistic model to fit the proportion of tips and nodes that belong to each lineage through time. This is done by using the function *estimate_rel_fitness_groups_with_branches*, which takes in entry:

- the *dataset_with_nodes* dataframe, with a column 'groups' which gives the group ID of each node in the dataset
- the timed tree
- *min_year*, the starting year at which to start fitting the model
- the window size (*window*), or number of windows (*N*), to divide the time series (from *min_year* to the last time point), compute proportions and fit the model.

For an example, see the SARS-CoV-2 code in Supplementary Text 4.

Lineage-defining mutations

Use the function *association_scores_per_group* to compute the association score of each mutation to each group. The function takes in entry:

- *dataset_with_nodes*
- *dataset_with_inferred_reconstruction*: a dataframe with the same first columns as *dataset_with_nodes* and then one column per snp its ancestral reconstruction along all nodes
- *tree*: timed tree
- *possible_snps*: the list of mutations that need to be considered
- *upstream_window*: for each group, how far upstream to consider mutations
- *downstream_window*: for each group, consider nodes from the MRCA up to this time

The codes to perform this analysis on the SARS-CoV-2 data is available in the file *2_4_Lineage_Defining_mutations.R*, in the folder *2_Functions*. As the SARS-CoV-2 genetic data is restricted, we cannot provide the raw data, but we provide all the sequence accession number to download them from GISAID.

Supplementary Text 4: Step-by-step guidance using the SARS-CoV-2 dataset as an example dataset.

This example, together with the codes, are also available in the README at https://zenodo.org/records/13952222 [Ref: [62]].

We provide here a working example on the SARS-CoV-2 timed phylogeny. You can go to specific sections of interest, however we recommend reading through all of this example.
Sections of this example:

- Load codes and SARS-CoV-2 data
- Compute the SARS-CoV-2 index dynamics
- Find SARS-CoV-2 clades based on index dynamics
- Quantify the fitness of detected SARS-CoV-2 lineage

Load codes and SARS-CoV-2 data

Load index functions

First, source all the necessary functions:

```
source(file = '2_Functions/2_1_Index_computation_20240909.R')
source(file = '2_Functions/2_2_Lineage_detection_20240909.R')
source(file = '2_Functions/2_3_Lineage_fitness_20240909.R')
```

Load necessary packages

```
library(ape, quiet = T); library(phytools, quiet = T); library(stringr, quiet = T)
library(MetBrewer, quiet = T); library(parallel, quiet = T); library(mgcv, quiet = T)
library(cowplot, quiet = T); library(ggplot2, quiet = T); library(ggtree, quiet = T);
library(cmdstanr, quiet = T); library(binom, quiet = T)
```

Versions:
Packages: ape v5.7-1, phytools v1.9-16, stringr v1.5.0, MetBrewer v0.2.0, parallel v4.1.2, mgcv v1.8-42, cowplot v1.1.1, ggplot2 v3.4.3, ggtree v3.2.1, cmdstanr v0.5.2, binom v1.1
R: 4.1.2

Load data

Load the NexStrain SARS-CoV-2 tree, in which all the tip name include: collection time, location and Pango lineage

```
tree_sars_cov2 =
read.nexus('1_Data/1_1_SARS_CoV_2/Tree_SARSCoV2_global_alltime_nextstrain_20230414.nexus')
## Make sure the tree is binary, and ladderized
tree_sars_cov2 = collapse.singles(ladderize(multi2di(tree_sars_cov2, random = F), right = F))
## Names all sequences
names_seqs = tree_sars_cov2$tip.label
n_seq = length(names_seqs)
## Collection times of all sequences
```

```
times_seqs = as.numeric(sapply(names_seqs, function(x)tail(str_split(x, pattern = '/')[[1]],2)[1]))
## Nextstrain clades of all sequences
clades_seqs = sapply(names_seqs, function(x)tail(str_split(x, pattern = '/')[[1]],1))
```

Compute the SARS-CoV-2 index dynamics

Index parameters

Set the index parameters.

```
## Length genome
genome_length = 29903 # reference nextstrain https://www.ncbi.nlm.nih.gov/nuccore/MN908947
## Mutation rate
mutation_rate = 8.1e-4 # mutation rate used by nextstrain https://github.com/nextstrain/ncov
## Parameters for the index
timescale = 0.15 ## Timescale
## Window of time on which to search for samples in the population
wind = 15 #days
wind = wind/365
```

Compute pairwise distance matrix

Compute distance between each pair of sequences and internal nodes in the tree

```
genetic_distance_mat = dist.nodes.with.names(tree_sars_cov2)
```

Get the time of each internal node

```
nroot = length(tree_sars_cov2$tip.label) + 1 ## Root number
distance_to_root = genetic_distance_mat[nroot,]
root_height = times_seqs[which(names_seqs == names(distance_to_root[1]))] - distance_to_root[1]
nodes_height = root_height + distance_to_root[n_seq+(1:(n_seq-1))]
```

Preparation data tips and nodes

Prepare the main dataframe, where the index and lineages of all nodes (internal and terminal) are going to be stored.

```
# Meta-data with all nodes
dataset_with_nodes = data.frame('ID' = c(1:n_seq, n_seq+(1:(n_seq-1))),
                     'name_seq' = c(names_seqs, n_seq+(1:(n_seq-1))),
                     'time' = c(times_seqs, nodes_height),
                     'is.node' = c(rep('no', n_seq), rep('yes', (n_seq-1))),
                     'Nextstrain_clade' = c(clades_seqs, rep(NA, n_seq-1)))
```

Compute index of every tip and node

```
dataset_with_nodes$index = compute.index(time_distance_mat = genetic_distance_mat,
                          timed_tree = tree_sars_cov2,
                          time_window = wind,
                          metadata = dataset_with_nodes,
```

```
                              mutation_rate = mutation_rate,
                              timescale = timescale,
                              genome_length = genome_length)
```

Plot tree & index below, with colors from NextStrain clades

First, generate the color key, based on the Nextstrain clade of each sequence.

```
## Color key for Nextstrain clades
colors_clade = met.brewer(name="Cross",
n=length(levels(as.factor(dataset_with_nodes$Nextstrain_clade))), type="continuous")

## Color of each node, based on the key
dataset_with_nodes$Nextstrain_clade_color = as.factor(dataset_with_nodes$Nextstrain_clade)
clade_labels = levels(dataset_with_nodes$Nextstrain_clade_color)
levels(dataset_with_nodes$Nextstrain_clade_color) = colors_clade
dataset_with_nodes$Nextstrain_clade_color =
as.character(dataset_with_nodes$Nextstrain_clade_color)
```

Then plot the tree and index:

```
par(mfrow = c(2,1), oma = c(0,0,0,0), mar = c(4,4,0,0))

min_year = 2020
max_year = 2023.5

## Tree
plot(tree_sars_cov2, show.tip.label = FALSE,
    edge.color = 'grey', edge.width = 0.25,
    x.lim = c(min_year, max_year)-root_height)
tiplabels(pch = 16, col = dataset_with_nodes$Nextstrain_clade_color, cex = 0.3)
axisPhylo_NL(side = 1, root.time = root_height, backward = F,
          at_axis = seq(min_year, max_year, 0.5)-root_height,
          lab_axis = seq(min_year, max_year, 0.5), lwd = 0.5)
## Index
plot(dataset_with_nodes$time,
    dataset_with_nodes$index,
    col = adjustcolor(dataset_with_nodes$Nextstrain_clade_color, alpha.f = 1),
    bty = 'n', xlim = c(min_year, max_year), cex = 0.4,
    pch = 16, bty = 'n', ylim = c(0, 1),
    main = paste0(''),
    ylab = 'Index', xlab = 'Time (years)', xaxt = 'n', yaxt = 'n')
axis(2, las = 2, lwd = 0.5)
axis(1, lwd = 0.5)

# Color key
legend('topright',
```

```
legend = clade_labels,
fill = colors_clade, border = colors_clade,
cex = 0.5, bty = 'n', ncol = 5)
```

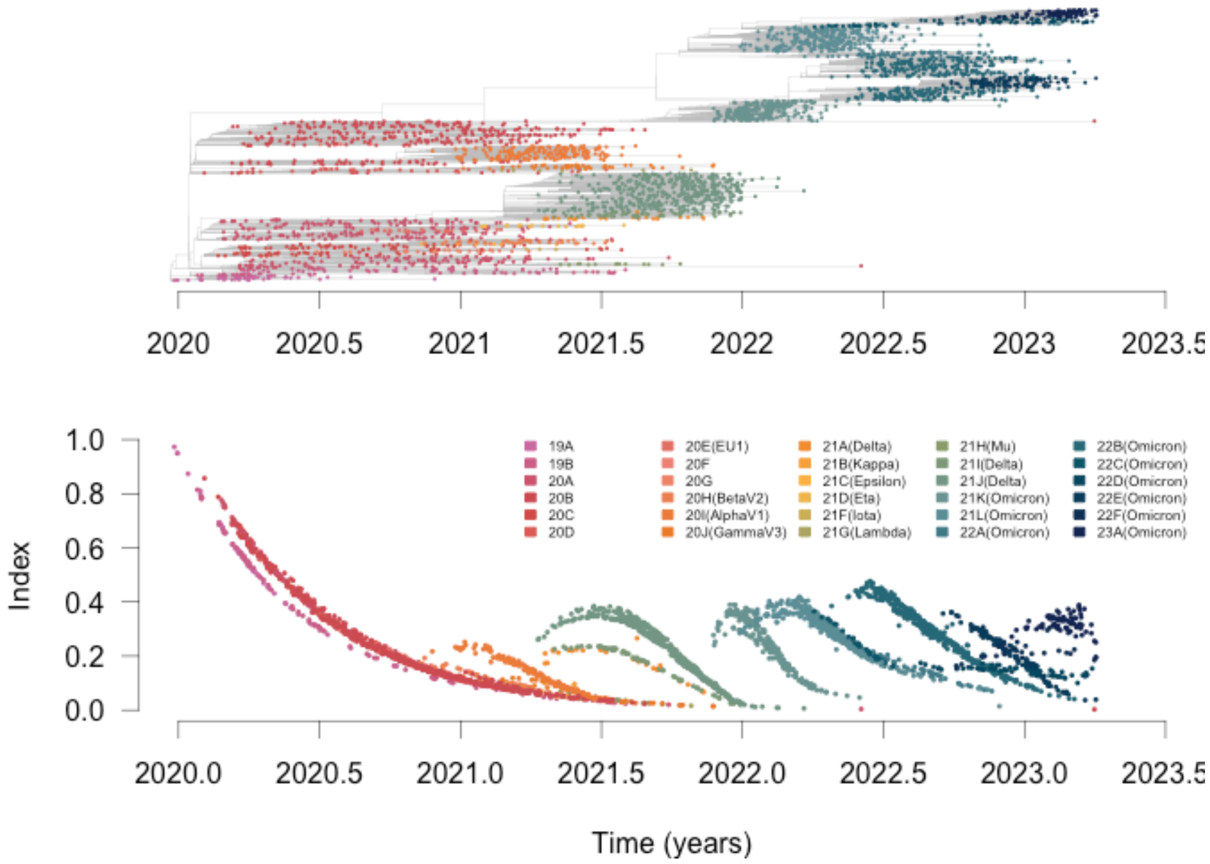

Find SARS-CoV-2 clades based on index dynamics

Run the lineage detection algorithm on SARS-CoV-2 data

Parameters for the detection:

```
time_window_initial = 2030;
time_window_increment = 100;
p_value_smooth = 0.05
weight_by_time = 0.1
k_smooth = -1
plot_screening = F
min_descendants_per_tested_node = 30
min_group_size = 30
weighting_transformation = c('inv_sqrt')

parallelize_code = T
number_cores = 2

max_stepwise_deviance_explained_threshold = 0
max_groups_found = 13
```

```
stepwise_AIC_threshold = 0

keep_track = T
```

Run the detection function (this steps takes approximately <10 min on 2 cores):

```
start_time = Sys.time()
potential_splits = find.groups.by.index.dynamics(timed_tree = tree_sars_cov2,
                                    metadata = dataset_with_nodes,
                                    node_support = tree_sars_cov2$edge.length[match((n_seq+1):(2*n_seq-
1), tree_sars_cov2$edge[,2])],
                                    threshold_node_support = 1/(29903*0.00081),
                                    time_window_initial = time_window_initial,
                                    time_window_increment = time_window_increment,
                                    min_descendants_per_tested_node =
min_descendants_per_tested_node,
                                    min_group_size = min_group_size,
                                    p_value_smooth = p_value_smooth,
                                    stepwise_deviance_explained_threshold =
max_stepwise_deviance_explained_threshold,
                                    stepwise_AIC_threshold = stepwise_AIC_threshold,
                                    weight_by_time = weight_by_time,
                                    weighting_transformation = weighting_transformation,
                                    k_smooth = k_smooth,
                                    parallelize_code = parallelize_code,
                                    number_cores = number_cores,
                                    plot_screening = plot_screening,
                                    max_groups_found = max_groups_found,
                                    keep_track = keep_track)
end_time = Sys.time()
print(end_time - start_time)
```

Instead, you may wish to load the results:

```
potential_splits = readRDS('README_files/potential_splits.rds')
```

Look at the deviance explained by the models with different number of groups.
Here for simplicity we directly chose max_groups_found = 13, which is the number of groups used in the original analysis. To decide on 13 groups, we initially ran the algorithm up to 30 groups.

```
df_explained_dev = data.frame('N_groups' = 0:length(potential_splits$best_dev_explained),
                    'Non_explained_deviance' = (1-c(potential_splits$first_dev,
potential_splits$best_dev_explained)),
                    'Non_explained_deviance_log' = log(1-c(potential_splits$first_dev,
potential_splits$best_dev_explained)))
```

```
df_explained_dev$Non_explained_deviance_log = df_explained_dev$Non_explained_deviance_log-
min(df_explained_dev$Non_explained_deviance_log)

par(mfrow = c(1,2), oma = c(2,2,1,1), mar = c(2,2,2,0.5), mgp = c(0.75,0.25,0), cex.axis=0.5,
cex.lab=0.5, cex.main=0.7, cex.sub=0.5)
plot(df_explained_dev$N_groups,
    df_explained_dev$Non_explained_deviance,
    bty = 'n', ylim = c(0, ceiling(10*max(df_explained_dev$Non_explained_deviance))/10),
    xaxt = 'n', yaxt = 'n', pch = 16, main = 'linear scale', cex = 0.5,
    ylab = 'Non-explained deviance (%)', xlab = 'Number of groups')
axis(1, lwd = 0.5, tck=-0.02)
axis(2, las = 2, at = seq(0,ceiling(10*max(df_explained_dev$Non_explained_deviance))/10,0.1),
    labels = seq(0, ceiling(10*max(df_explained_dev$Non_explained_deviance))/10,0.1)*100, lwd =
0.5, tck=-0.02)

plot(df_explained_dev$N_groups,
    (df_explained_dev$Non_explained_deviance),
    log = 'y',
    ylim = c(0.01, 1),
    bty = 'n',
    xaxt = 'n', yaxt = 'n', pch = 16, main = 'log scale', cex = 0.5,
    ylab = 'Non-explained deviance (%) - log scale', xlab = 'Number of groups')
axis(1, lwd = 0.5, tck=-0.02)
axis(2, las = 2, at = c(0.01, 0.1, 0.25, 0.5, 1),
    labels = c(0.01, 0.1, 0.25, 0.5, 1)*100, lwd = 0.5, tck=-0.02)
```

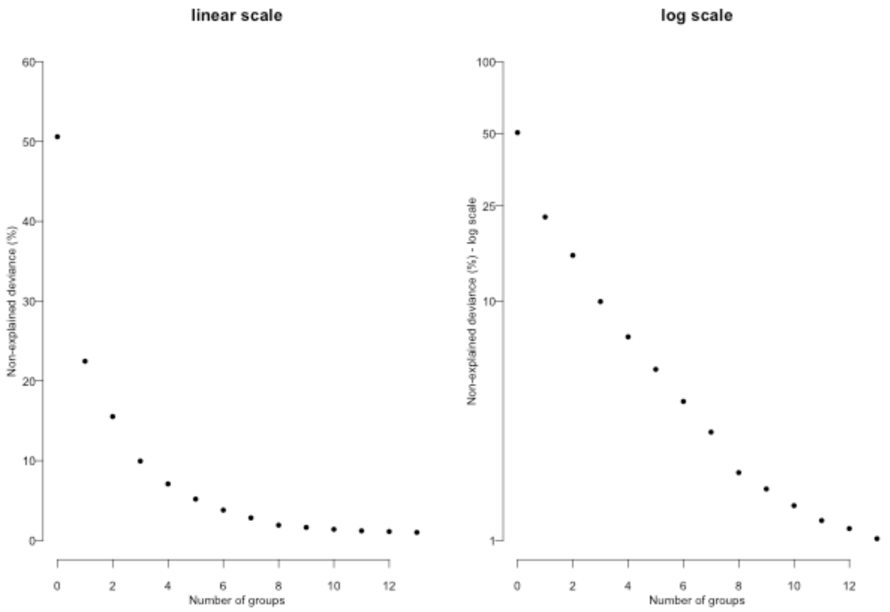

Optimize the number of groups: set the minimum number of sequences per group to 30, with a minimum frequency of 1%.

```
split = merge.groups(timed_tree = tree_sars_cov2, metadata = dataset_with_nodes,
                 initial_splits = potential_splits$potential_splits,
                 group_count_threshold = 30, group_freq_threshold = 0.01)
```

Label sequences with these new groups, and assign a color to each of them.

```
## Label sequences with new groups
dataset_with_nodes$groups = as.factor(split$groups)
## Reorder labels by time of emergence
name_groups = levels(dataset_with_nodes$groups)
time_groups_world = NULL
for(i in 1:length(name_groups)){
  time_groups_world = c(time_groups_world,
min(dataset_with_nodes$time[which(dataset_with_nodes$groups == name_groups[i] &
                                                  dataset_with_nodes$is.node == 'no')]))
}
levels(dataset_with_nodes$groups) = match(name_groups, order(time_groups_world, decreasing =
T))
dataset_with_nodes$groups = as.numeric(as.character(dataset_with_nodes$groups))
dataset_with_nodes$groups = as.factor(dataset_with_nodes$groups)
## Update names in split list
split$tip_and_nodes_groups = match(split$tip_and_nodes_groups, order(time_groups_world,
decreasing = T))
names(split$tip_and_nodes_groups) = 1:length(split$tip_and_nodes_groups)
split$groups = as.factor(split$groups)
levels(split$groups) = match(name_groups, order(time_groups_world, decreasing = T))
split$groups = as.numeric(as.character(split$groups))
## Choose color palette
n_groups <- length(name_groups)
colors_groups = (met.brewer(name="Cross", n=n_groups, type="continuous"))
## Color each group
dataset_with_nodes$group_color = dataset_with_nodes$groups
levels(dataset_with_nodes$group_color) = colors_groups
dataset_with_nodes$group_color = as.character(dataset_with_nodes$group_color)
```

Plot tree & index below, with colors from index-defined groups

Plot the tree and index colored with the new groups:

```
par(mfrow = c(2,1), oma = c(0,0,0,0), mar = c(4,4,0,0))

## Tree
plot(tree_sars_cov2, show.tip.label = FALSE,
     edge.color = 'grey', edge.width = 0.25,
```

```
    x.lim = c(min_year, max_year)-root_height)
tiplabels(pch = 16, col = dataset_with_nodes$group_color, cex = 0.3)
axisPhylo_NL(side = 1, root.time = root_height, backward = F,
          at_axis = seq(min_year, max_year, 0.5)-root_height,
          lab_axis = seq(min_year, max_year, 0.5), lwd = 0.5)

## Index colored by group
plot(dataset_with_nodes$time,
    dataset_with_nodes$index,
    col = adjustcolor(dataset_with_nodes$group_color, alpha.f = 1),
    bty = 'n', xlim = c(min_year, max_year), cex = 0.5,
    pch = 16, bty = 'n', #ylim = c(0, 1),
    main = paste0(''), #log = 'y',
    ylab = 'Index', xlab = 'Time (years)', yaxt = 'n')
axis(2, las = 2)
# Color key
legend('topright',
      legend = name_groups,
      fill = colors_groups, border = colors_groups,
      cex = 0.5, bty = 'n', ncol = 5)
```

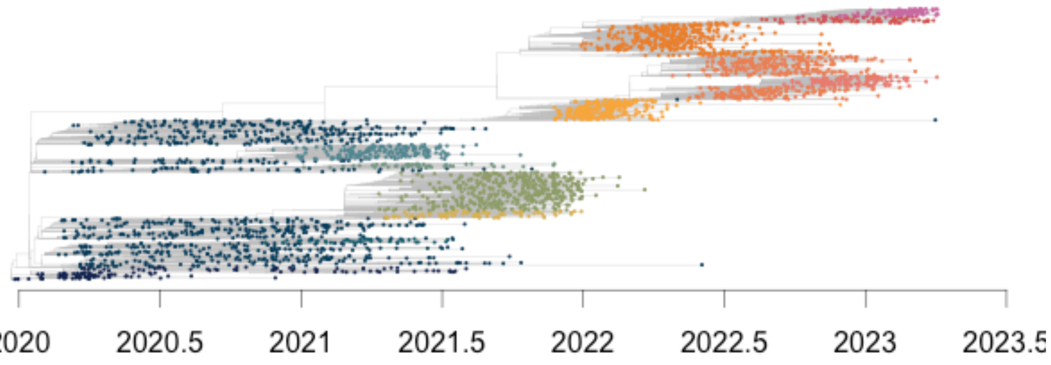

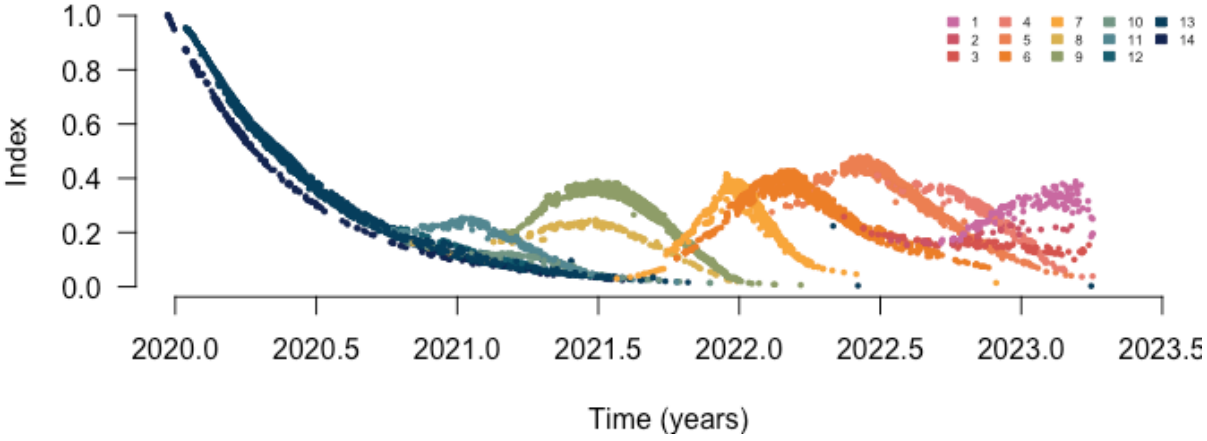

Compare NextStrain groups and groups called with the index

Generate SARS-CoV-2 trees coloured with each set of groups next to each other:

```
## Tree with index-defined groups
groups = matrix(dataset_with_nodes$groups[which(dataset_with_nodes$is.node == 'no')], ncol = 1)
colnames(groups) = 'groups'
rownames(groups) = dataset_with_nodes$name_seq[which(dataset_with_nodes$is.node == 'no')]
cols = as.character(colors_groups)
names(cols) = as.character(1:max(as.numeric(name_groups)))
plot_tree_sars_world_groups <- ggtree(tree_sars_cov2,
mrsd=lubridate::date_decimal(max(times_seqs)), size = 0.10,
                aes(color = as.character(dataset_with_nodes$groups))) +
  scale_color_manual(values = cols)+theme_tree2()
plot_tree_sars_world_groups = gheatmap(plot_tree_sars_world_groups, groups, offset=0.1,
width=0.10,
                 colnames=FALSE, legend_title="Group", color=NA) +
  scale_fill_manual(values = (cols))+scale_y_continuous(expand=c(0, 0.3))+theme(legend.position =
'none')

## Tree with NextStrain clades
Nextstrain = matrix(dataset_with_nodes$Nextstrain_clade[which(dataset_with_nodes$is.node ==
'no')], ncol = 1)
colnames(Nextstrain) = 'groups'
rownames(Nextstrain) = dataset_with_nodes$name_seq[which(dataset_with_nodes$is.node ==
'no')]
cols_NextStrain = as.character(colors_clade)
names(cols_NextStrain) = clade_labels
plot_tree_sars_world_Nextstrain <- ggtree(tree_sars_cov2,
mrsd=lubridate::date_decimal(max(times_seqs)), size = 0.10,
                                aes(color = as.character(dataset_with_nodes$Nextstrain_clade))) +
  scale_color_manual(values = cols_NextStrain)+
  theme_tree2(legend = 'none')
plot_tree_sars_world_Nextstrain = gheatmap(plot_tree_sars_world_Nextstrain, Nextstrain,
offset=0.1, width=0.10,
                  colnames=FALSE, legend_title="Group", color=NA) +
  scale_fill_manual(values = cols_NextStrain, na.value = 'white')+
  scale_x_reverse() +
  scale_y_continuous(expand=c(0, 0.3))+
  theme(legend.position = 'none')
```

Plot the generated SARS-CoV-2 trees:

```
plot_grid(plot_tree_sars_world_groups, plot_tree_sars_world_Nextstrain,
        rel_widths = c(1, 1), labels = c('Automatic clades', 'NextStrain clades'), label_size = 10, label_x =
c(0.1, 0.25), ncol = 2)
```

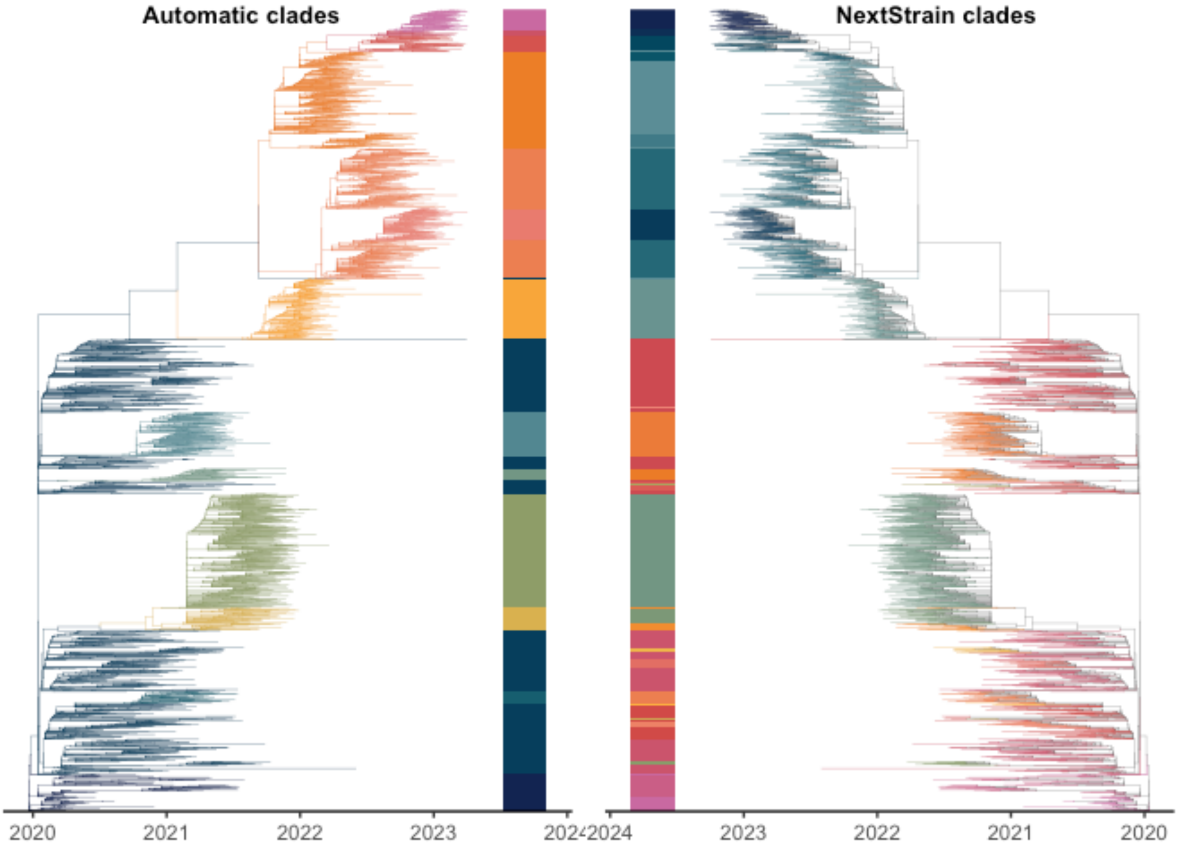

Quantify the fitness of detected SARS-CoV-2 lineages

Run the fitness model

Quantify the fitness of each group you can run the code (this steps takes approximately <5 min on 3 cores):

```
start_time = Sys.time()
## Load and compile stan code (this can take a few minutes)
model_compiled <- cmdstan_model(stan_file =
'2_Functions/Model_multinomial_logistic_birthdeath_lineage_fitness_20231220.stan')
## Run model on SARS-CoV-2 groups
res_fitness = estimate_rel_fitness_groups_with_branches(dataset_with_nodes =
dataset_with_nodes,
                              tree = tree_sars_cov2,
                              min_year = 2020,
                              window = 30/365,
                              model_compiled = model_compiled,
                              iter_warmup = 250, iter_sampling = 500, refresh = 50, seed = 1)
end_time = Sys.time()
print(end_time - start_time)
```

You might encounter a warning saying that '*alpha_true_GA*' has a missing init value - this is normal as those groups (ancestral groups that are not present at the start of the time series) do not always exist and therefore there is no default initial value. This is does not impact the model run. The seed has been set to 1 so allow for reproducible results.

To save some time, you may wish to load the results:
res_fitness = readRDS('README_files/res_fitness.rds')

Plot the fits and estimated parameters

Plot the fits:
order_colors = order(as.numeric(split$tip_and_nodes_groups))
colour_lineage = colors_groups[match(split$tip_and_nodes_groups[order_colors], name_groups)]

```
plot_fit_data_new(data = res_fitness$data,
        Chains = res_fitness$chains,
        colour_lineage = colour_lineage,
        xmin = 2020, xmax = 2023.5)
```

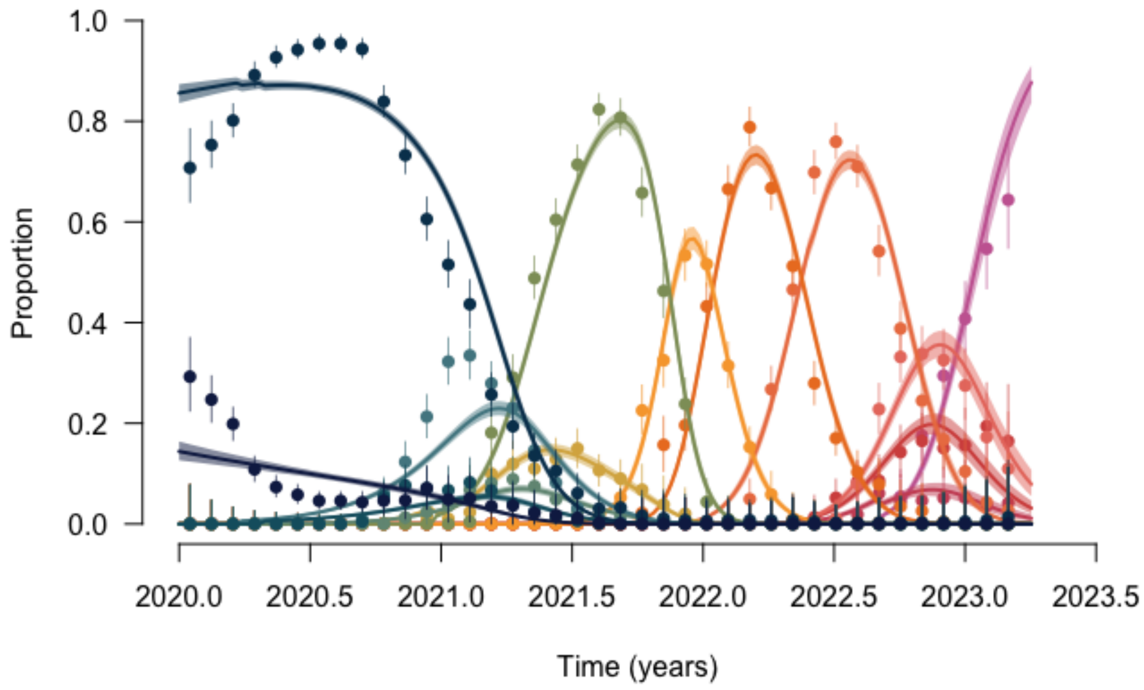

Supplementary references

63. Stadler, T. Simulating trees with a fixed number of extant species. *Syst. Biol.* **60**, 676–684 (2011).
64. Vaughan, T. G. ReMASTER: improved phylodynamic simulation for BEAST 2.7. *Bioinformatics* **40**, (2024).
65. Ly-Trong, N., Naser-Khdour, S., Lanfear, R. & Minh, B. Q. AliSim: A Fast and Versatile Phylogenetic Sequence Simulator for the Genomic Era. *Mol. Biol. Evol.* **39**, (2022).
66. Volz, E. M., Koelle, K. & Bedford, T. Viral phylodynamics. *PLoS Comput. Biol.* **9**, e1002947 (2013).

Supplementary figures

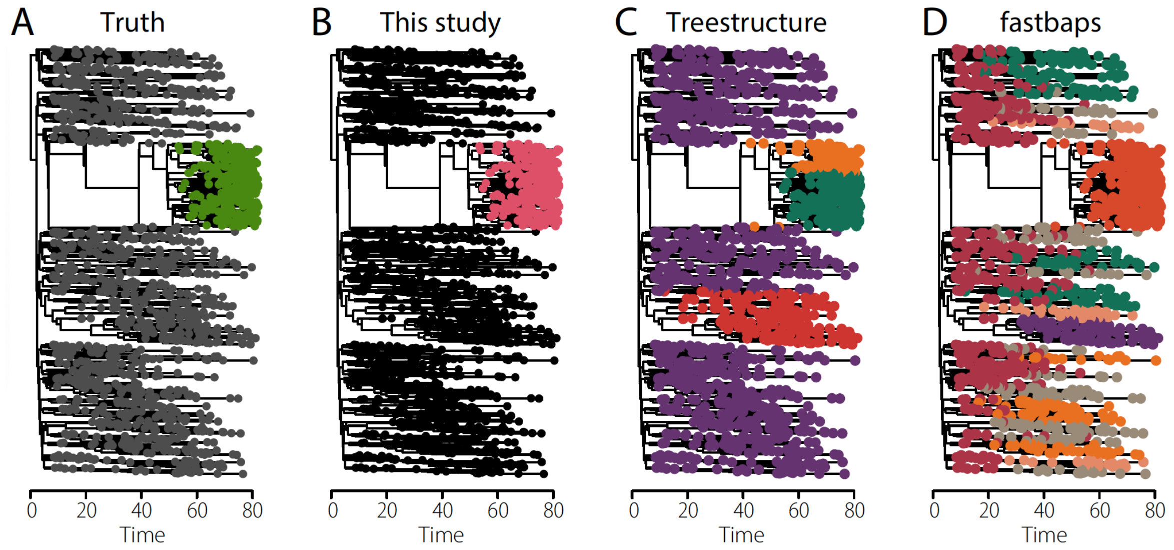

Supplementary Figure 1: Example of classification with our method, treestructure and fastbaps. We present an example of results on one simulated tree consisting of a background population (grey) and one emerging lineage (green) with a fitness advantage of 0.2 per time unit **(A)**. The results with our method are presented in **(B),** treestructure[15] in **(C)**, and fastbaps[14] in **(D).** For each method, the colours indicate the different lineages identified.

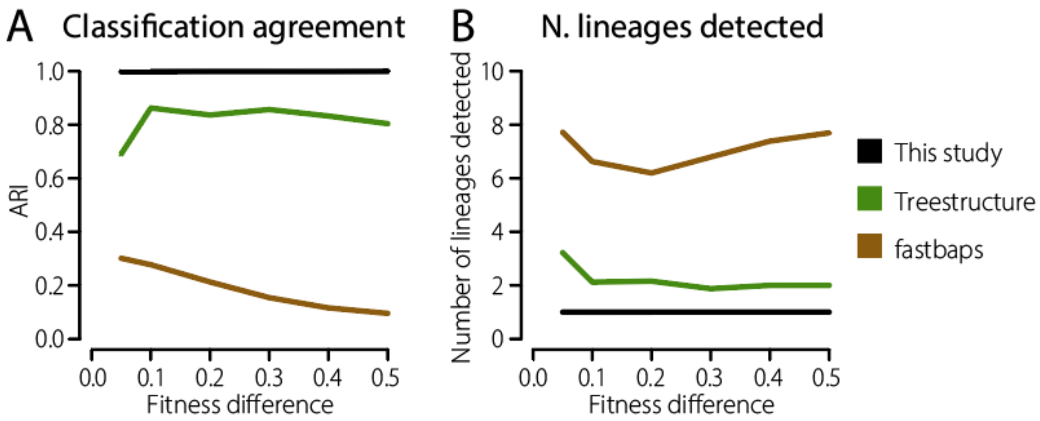

Supplementary Figure 2: Comparison of the lineages found by our method, treestructure and fastbaps on simulated datasets.

For each scenario and each method, we plot the classification agreement (Adjusted Rand Index [21]) **(A)**, and the number of lineages detected **(B)**. Colours denote the different methods.

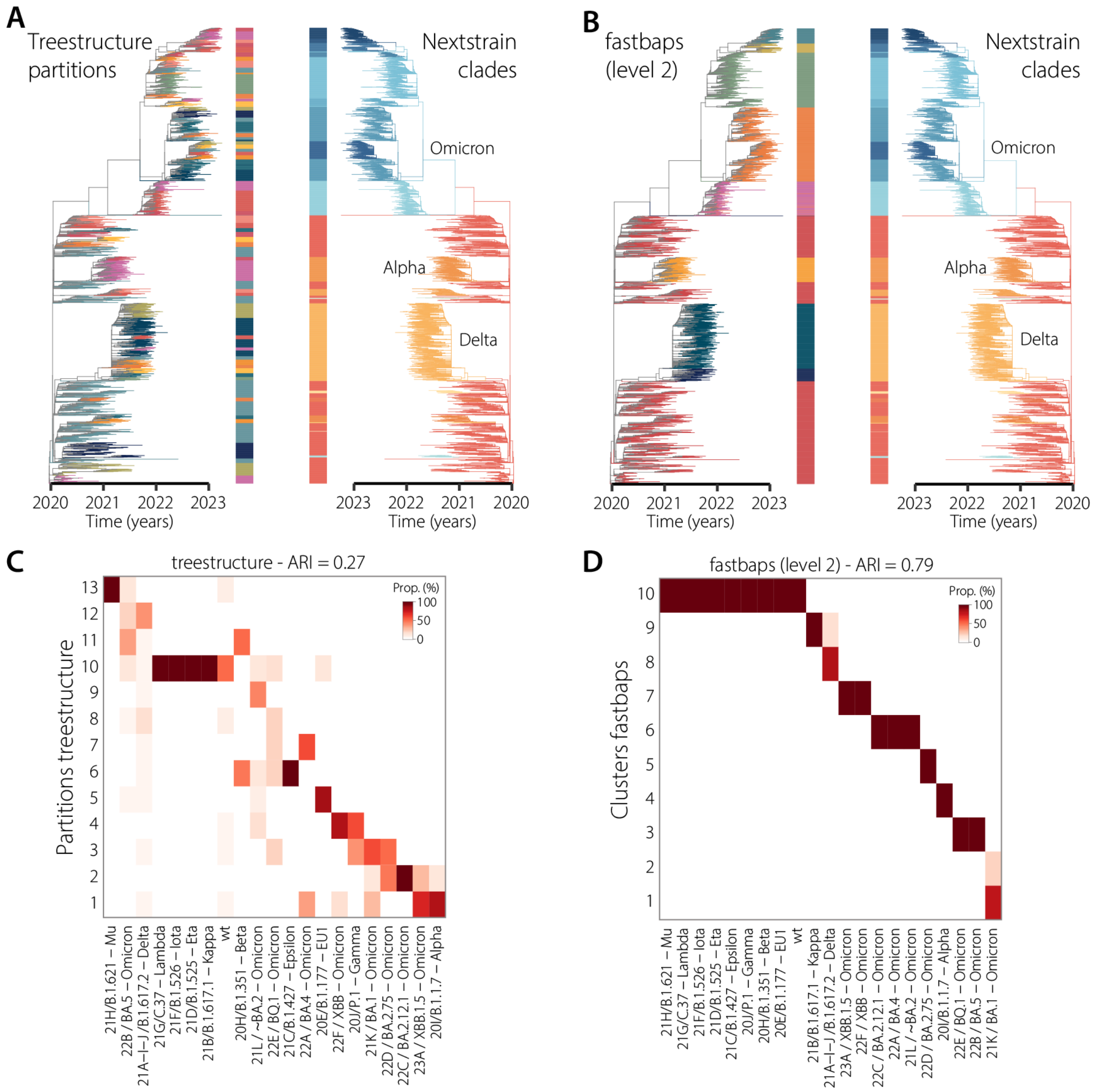

Supplementary Figure 3: SARS-CoV-2 lineages identified with treestructure and fastbaps

(A-B) Global SARS-CoV-2 trees coloured by the lineages identified with treestructure (A), or fastbaps (B). **(C-D)** We compare the lineages identified with either algorithm (y-axis) to the NextStrain clades (x-axis). Darker colours represent more agreement between both namings.

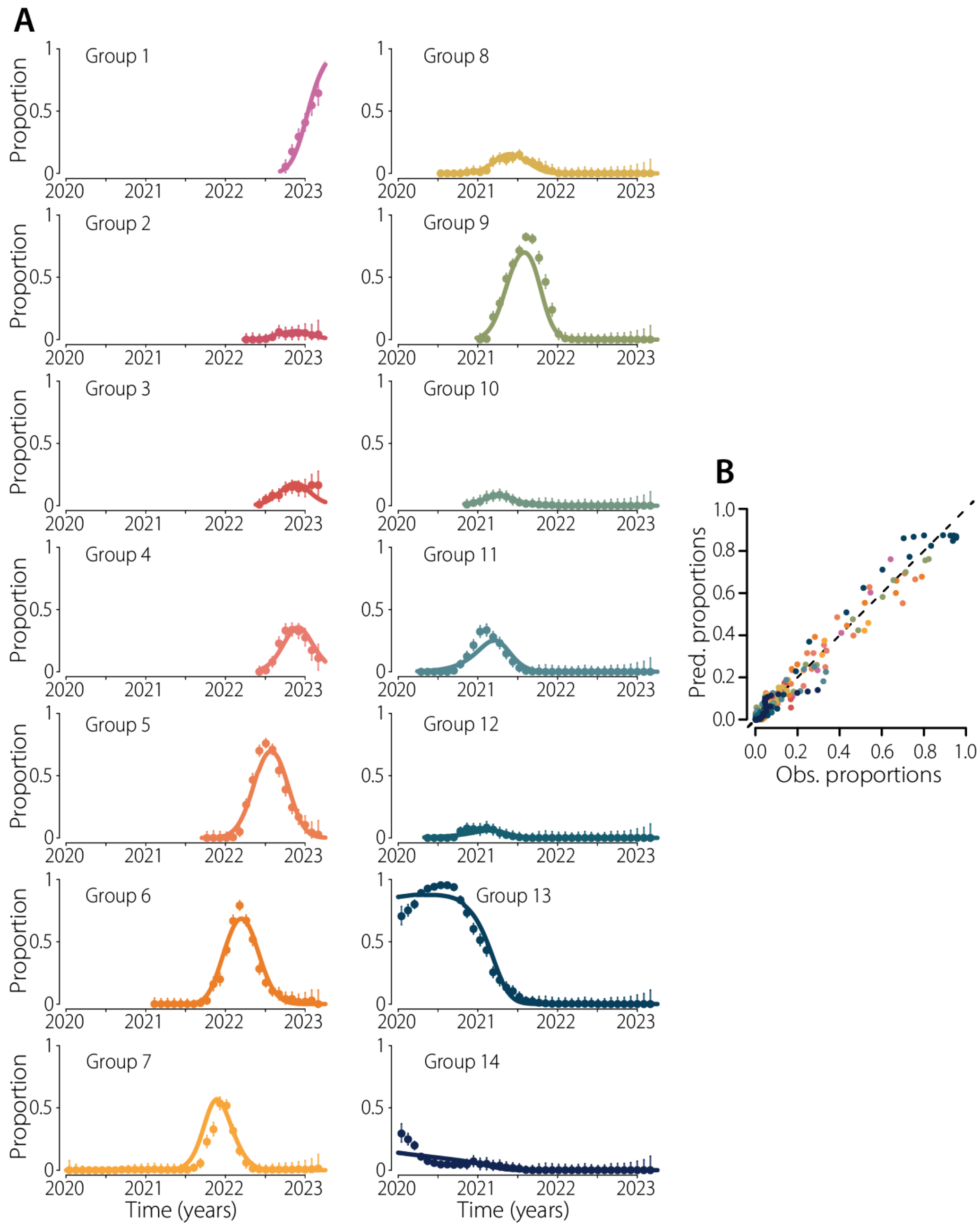

Supplementary Figure 4: Fitness model fits for all lineages of SARS-CoV-2

(A) Fits of the proportion of all the SARS-CoV-2 lineages. Coloured dots represent data, bars denote 95% confidence intervals. Coloured lines and shaded areas represent the median and 95% credible interval of the posterior. **(B)** Predicted versus observed proportions.

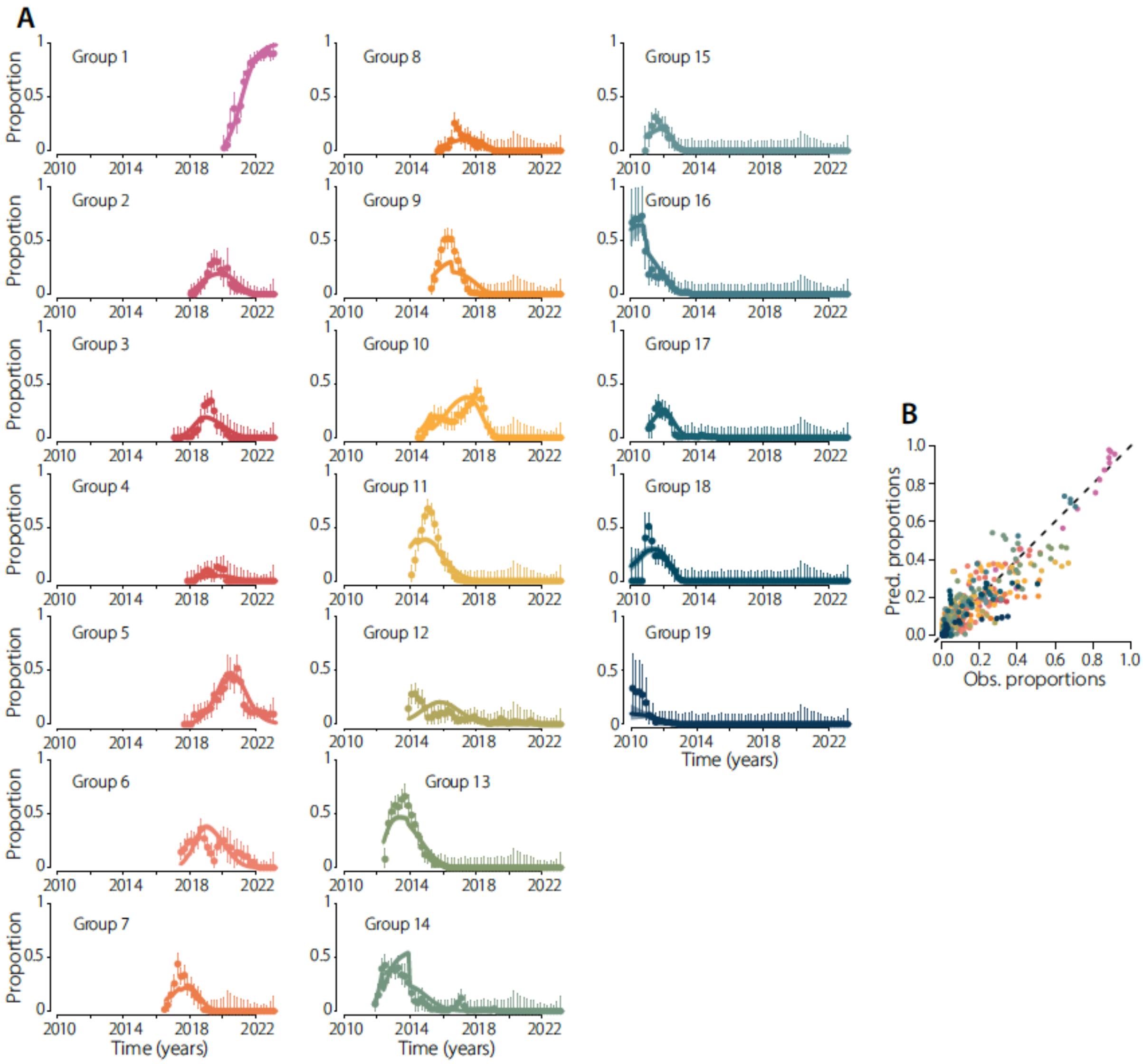

Supplementary Figure 5: Fitness model fits for all lineages of H3N2

(A) Fits of the proportion of all the H3N2 lineages. Coloured dots represent data, bars denote 95% confidence intervals. Coloured lines and shaded areas represent the median and 95% credible interval of the posterior. **(B)** Predicted versus observed proportions.

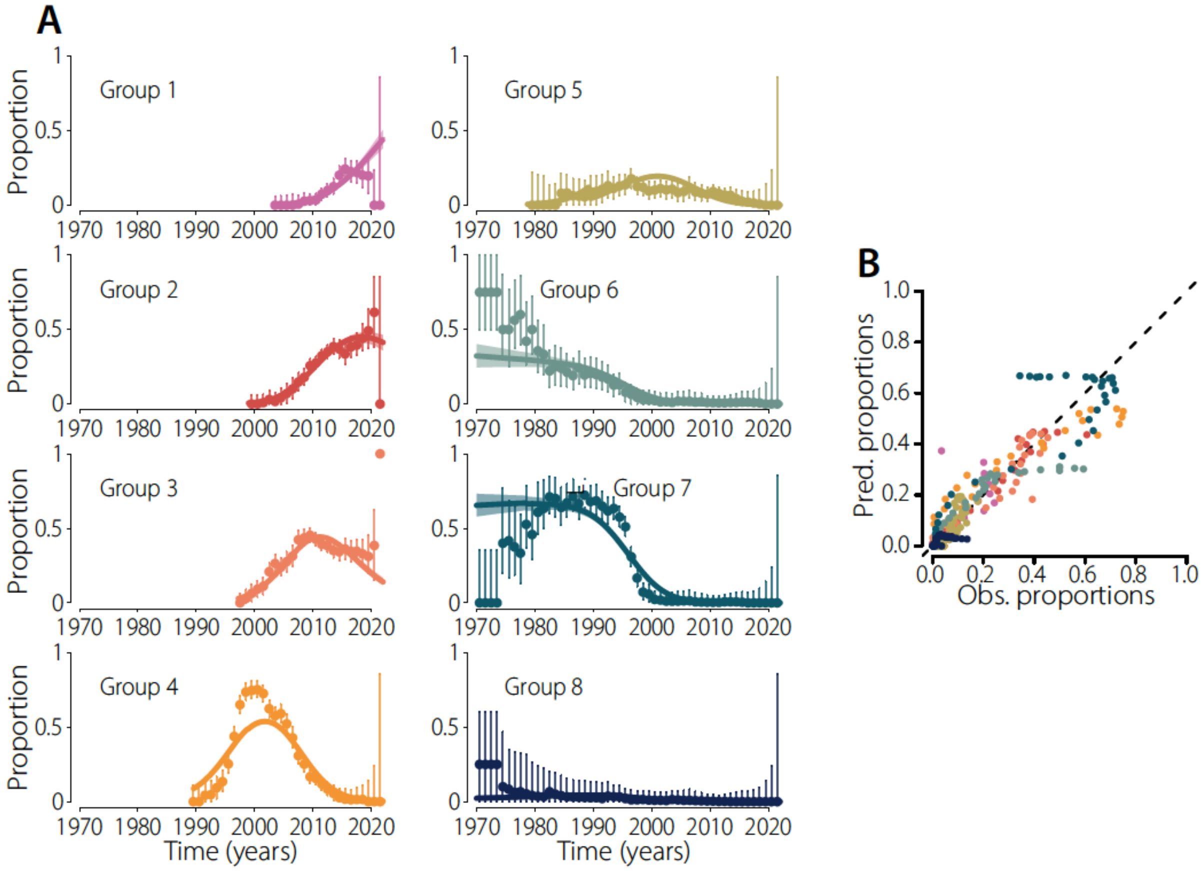

Supplementary Figure 6: Fitness model fits for all lineages of *B. pertussis*

(A) Fits of the proportion of all the *B. pertussis* lineages. Coloured dots represent data, bars denote 95% confidence intervals. Coloured lines and shaded areas represent the median and 95% credible interval of the posterior. **(B)** Predicted versus observed proportions.

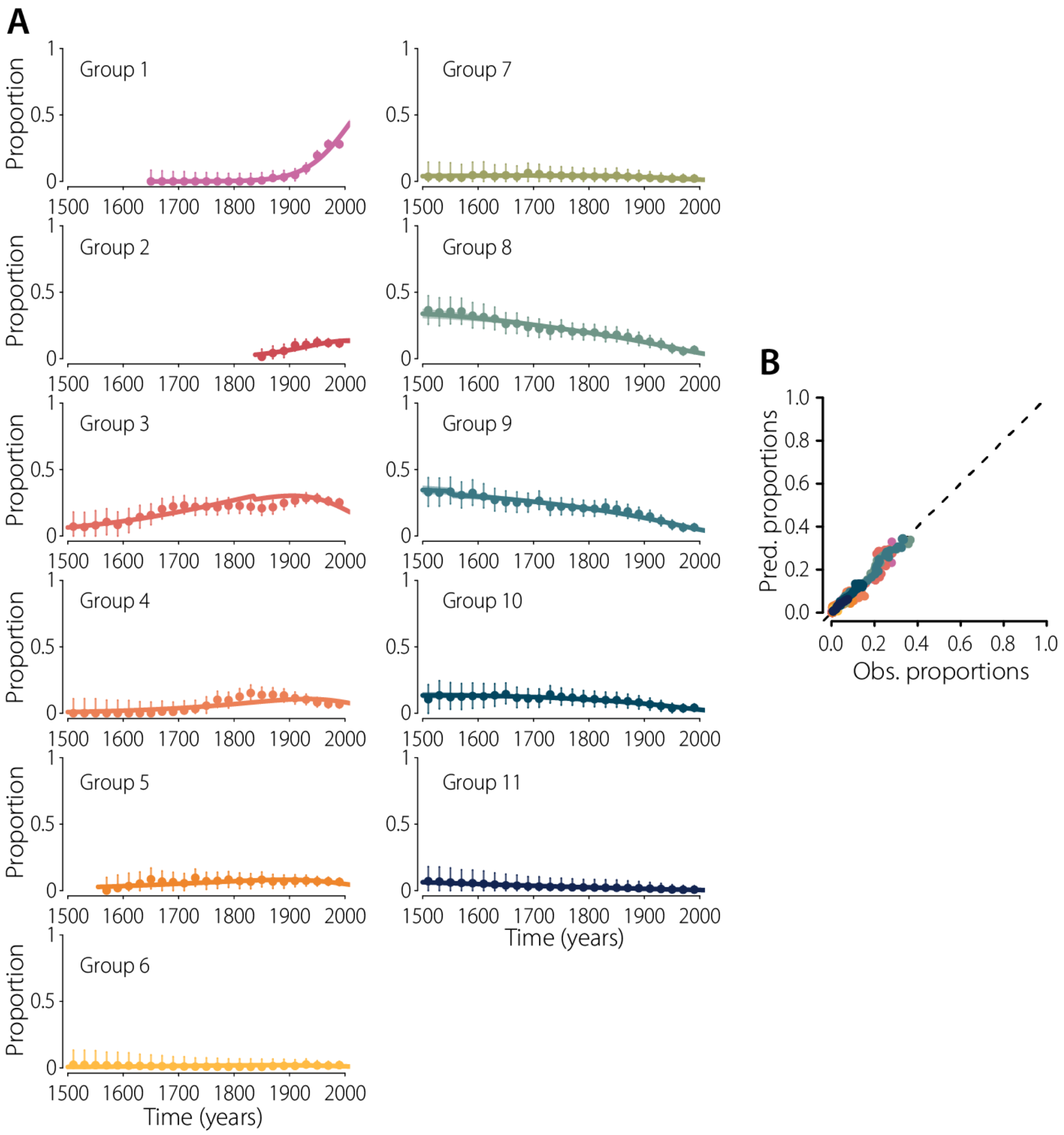

Supplementary Figure 7: Fitness model fits for all lineages of *M. tuberculosis.*

(A) Fits of the proportion of all the *M. tuberculosis* lineages. Coloured dots represent data, bars denote 95% confidence intervals. Coloured lines and shaded areas represent the median and 95% credible interval of the posterior. **(B)** Predicted versus observed proportion

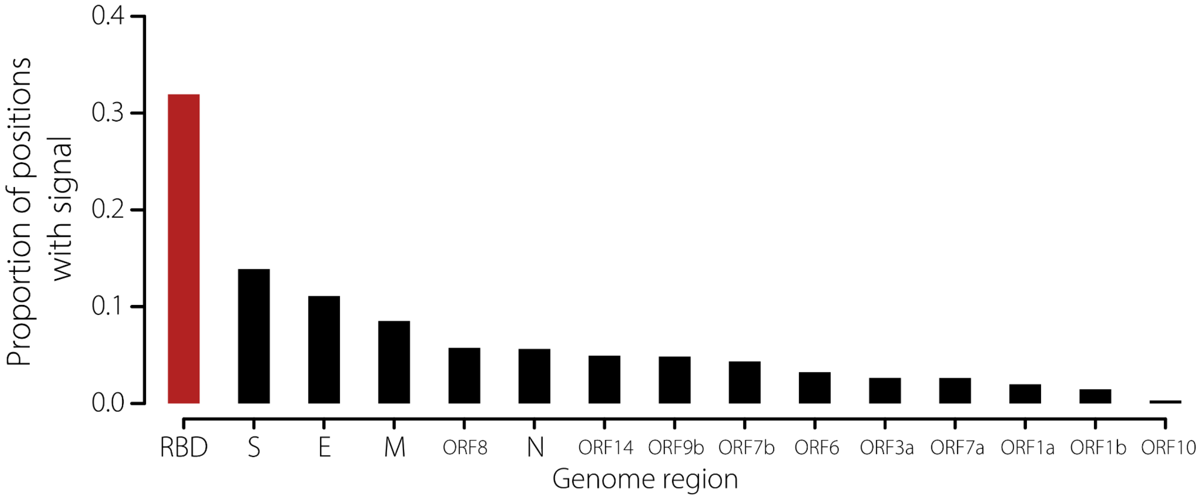

Supplementary Figure 8: Proportion of mutations that are defining the lineages of SARS-CoV-2 worldwide, by ORFs

Additionally to Figure 4E, we plot the proportion of amino acid substitutions that are lineage-defining within SARS-CoV-2 ORFs, and the Receptor Binding Domain (RBD).

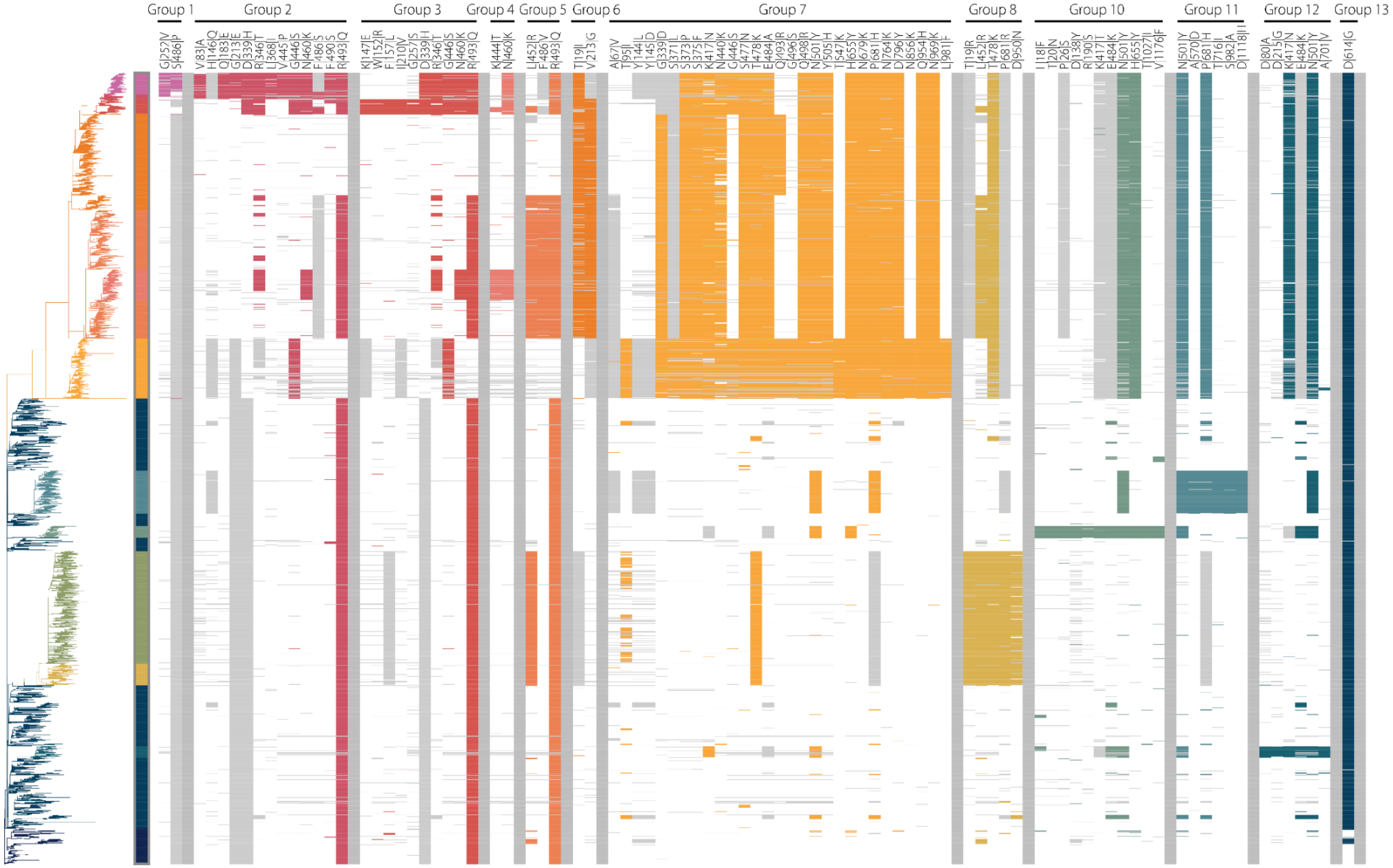

Supplementary Figure 9: Phylogenetic tree and mutations in the spike protein that are defining lineages in the global SARS-CoV-2 dataset

We present the SARS-CoV-2 time-resolved tree (left), together with the mutations that we found to be defining its lineages (right). Colours represent the different lineages. Each column on the right displays one mutation, with its name at the top. Colours denote isolates that are carrying the labelled mutation, white denotes the absence of that mutation (although isolates could have other mutations at this position), grey denotes an unknown amino acid. Some mutations (e.g., T478K or N501Y) are defining multiple lineages and are therefore plotted twice. The list of lineage-defining mutations can be found in Data File S5.

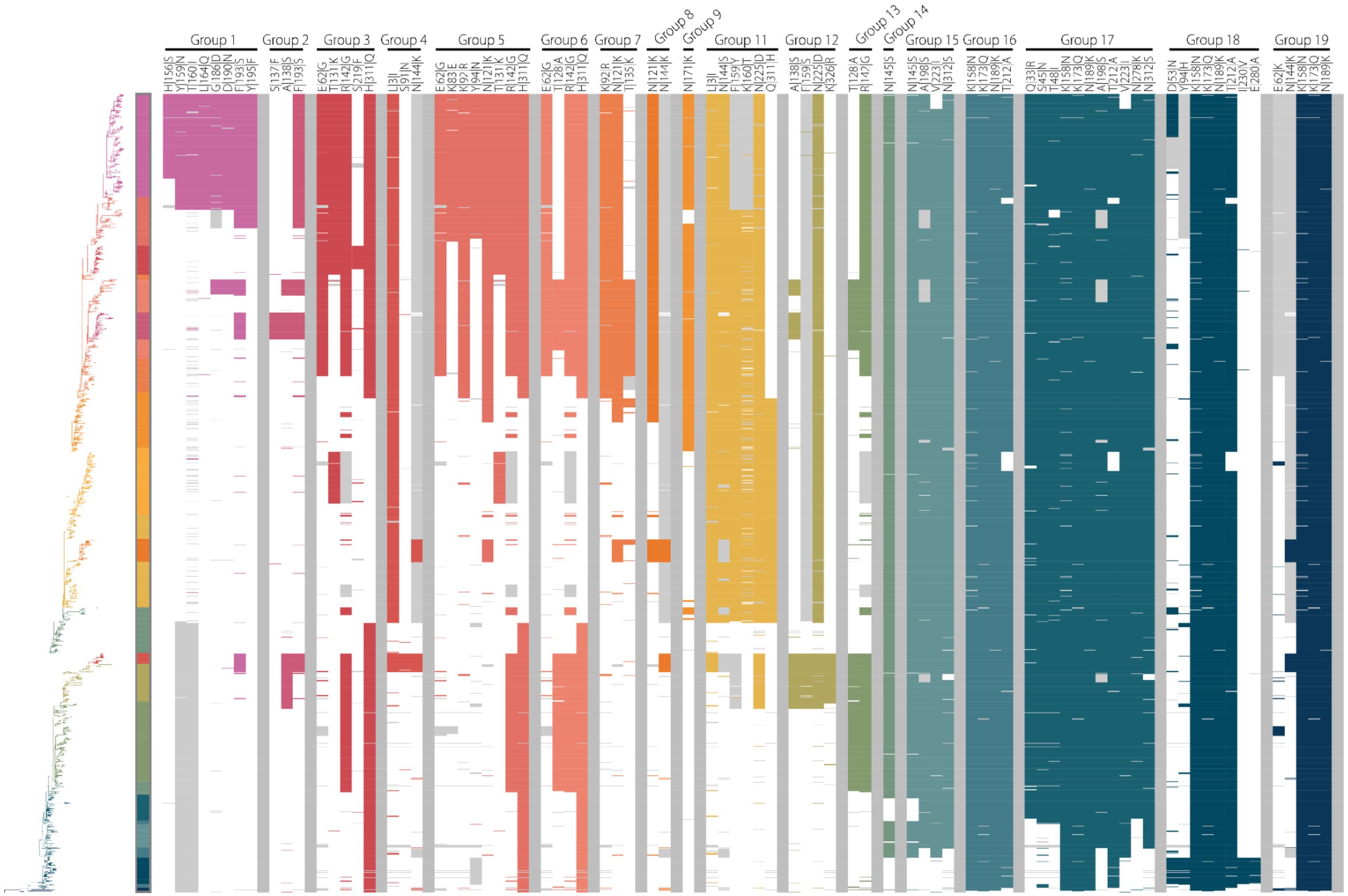

Supplementary Figure 10: Phylogenetic tree and mutations in the HA1 subunit that are defining lineages in the global H3N2 dataset
We present the H3N2 time-resolved tree (left), together with the mutations that we found to be defining its lineages (right). Colours represent the different lineages. Each column on the right displays one mutation, with its name at the top. Colours denote isolates that are carrying the labelled mutation, white denotes the absence of that mutation (although isolates could have other mutations at this position), grey denotes an unknown amino acid. Some mutations (e.g., N144K or F193S) are defining multiple lineages and are therefore plotted twice. The list of lineage-defining mutations can be found in Data File S6.

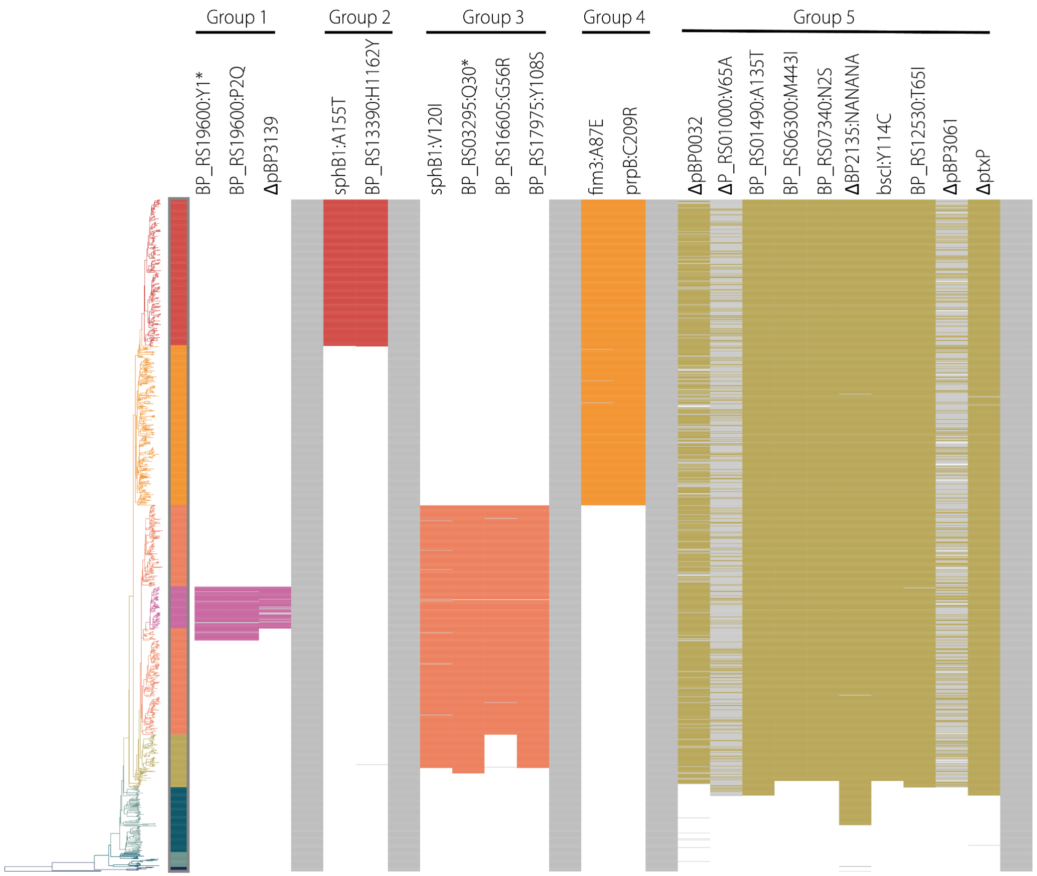

Supplementary Figure 11: Phylogenetic tree and mutations defining lineages in the *B. pertussis* dataset from in France

We present the *B. pertussis* time-resolved tree (left), together with the mutations that we found to be defining its lineages (right). Colours represent the different lineages. Each column on the right displays one mutation, with its name at the top. Colours denote isolates that are carrying the labelled mutation, white denotes the absence of that mutation (although isolates could have other mutations at this position), grey denotes an unknown nucleotide. The list of lineage-defining mutations can be found in Data File S7.

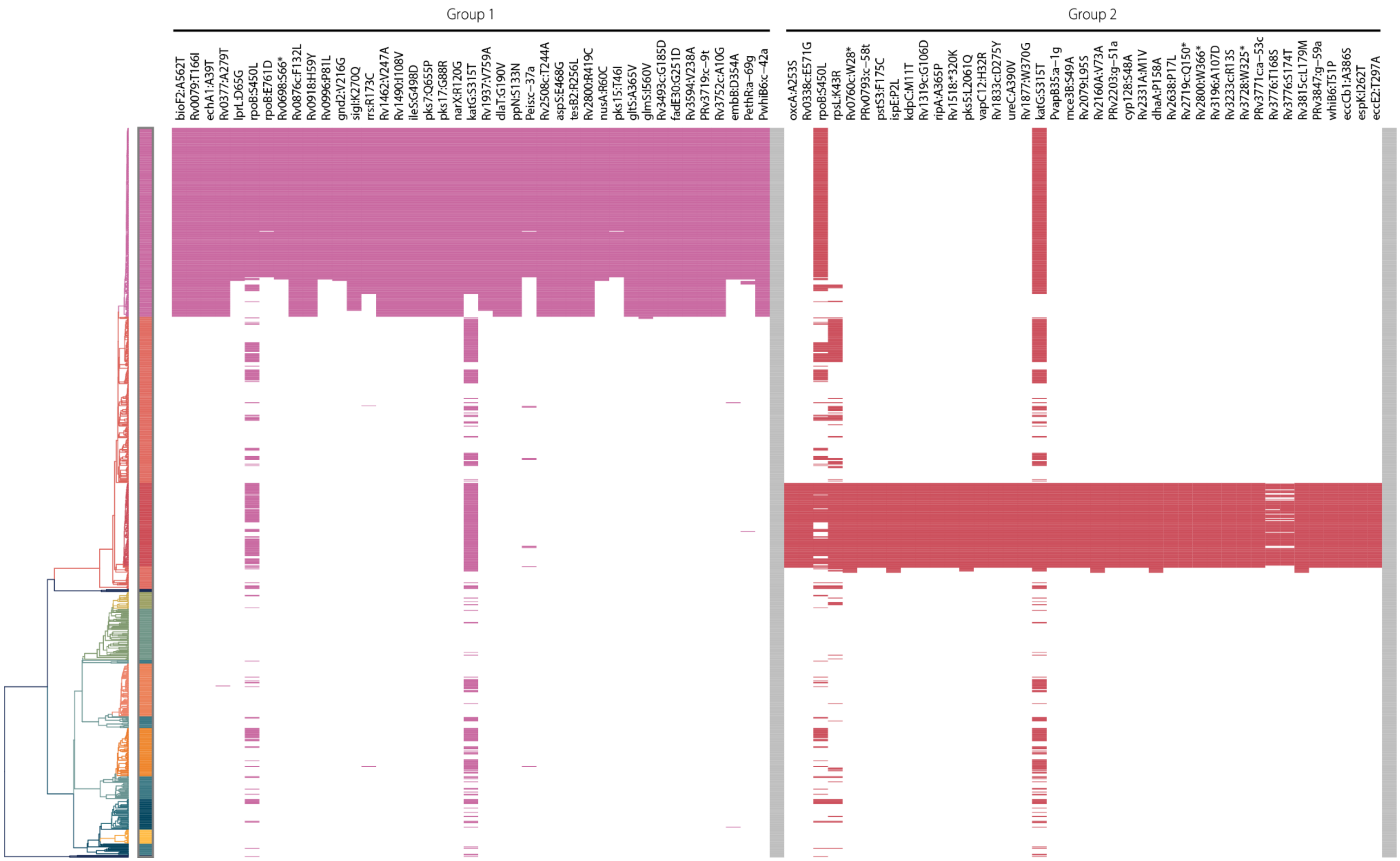

Supplementary Figure 12: Phylogenetic tree and mutations defining lineages 1 and 2 in the *M. tuberculosis* dataset from in Samara, Russia

We present the *M. tuberculosis* time-resolved tree (left), together with the mutations that we found to be defining the lineages 1 and 2 (right). Colours represent the different lineages. Each column on the right displays one mutation, with its name at the top. Colours denote isolates that are carrying the labelled mutation, white denotes the absence of that mutation (although isolates could have other mutations at this position). Some mutations (e.g., rpoB:S450L or katG:S315T) are defining both lineages and are therefore plotted twice. The list of lineage-defining mutations can be found in Data File S8.

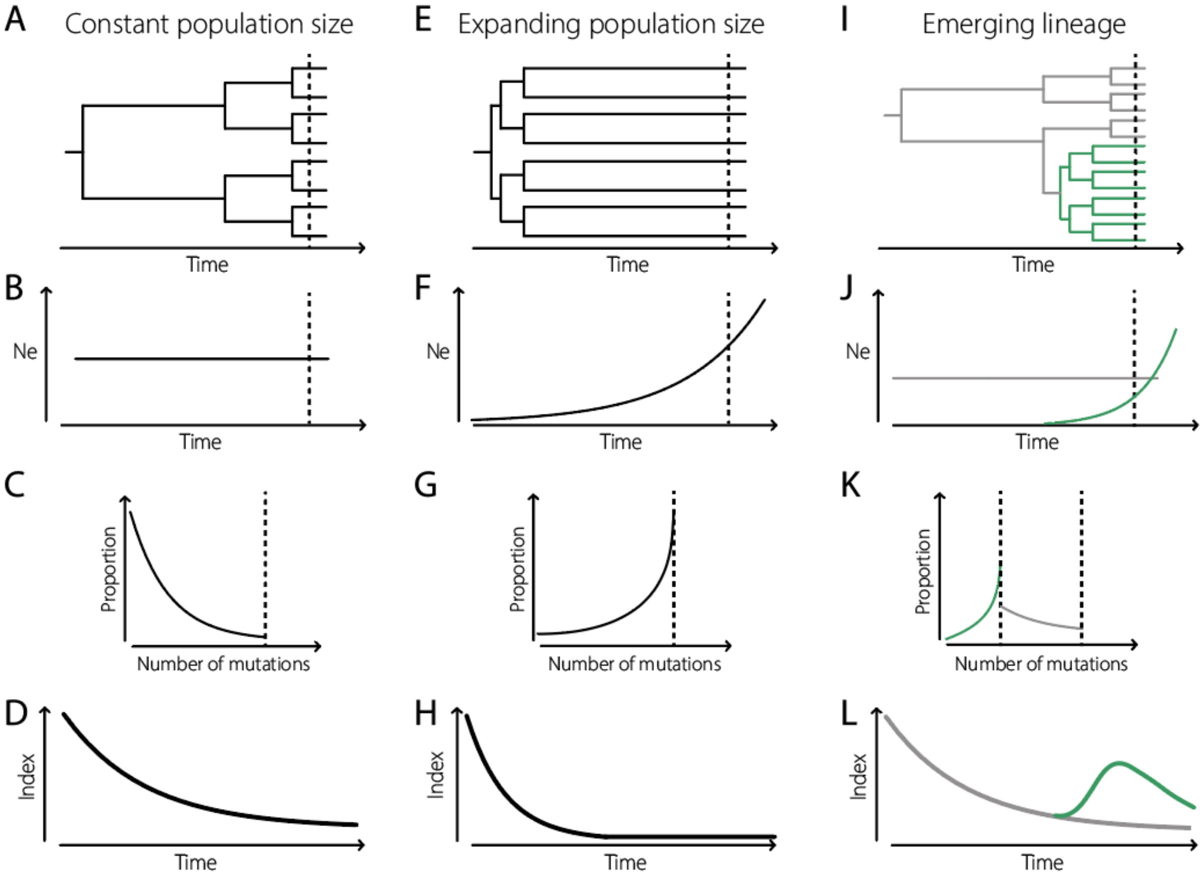

Supplementary Figure 13: Population history, pairwise distance distribution and index dynamics. **(A-D)** Constant effective population size. **(E-H)** Exponential population size. (A and B are inspired by Volz and colleagues, 2013[66]) **(I-L)** Case of an emerging, exponentially growing, lineage in a population of constant effective size.

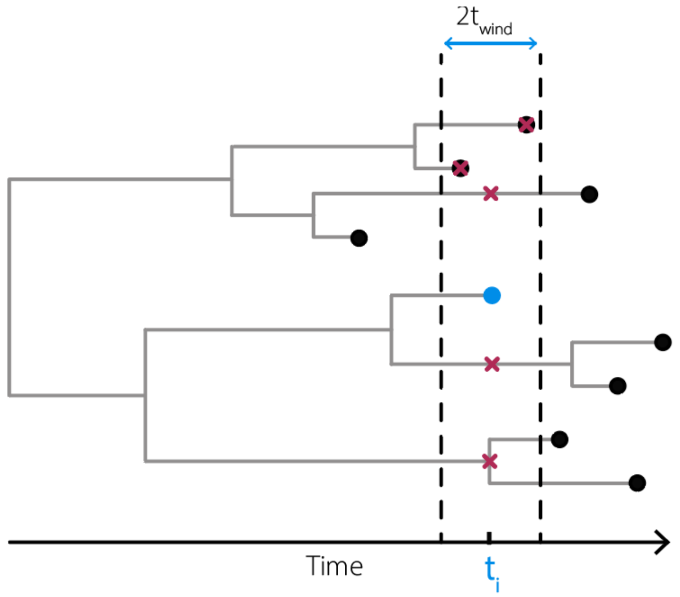

Supplementary Figure 14: Schematic of the notations used to compute the index on a timed-tree with sequences sampled through time.

The blue dot denotes the sequence of interest i, with t_i its sampling time. The dashed lines represent the window $[t_i - t_{wind};\ t_i + t_{wind}]$. All the nodes that fall within this window are considered to be circulating at the same time as i. These nodes are denotes by red crosses on the figure.

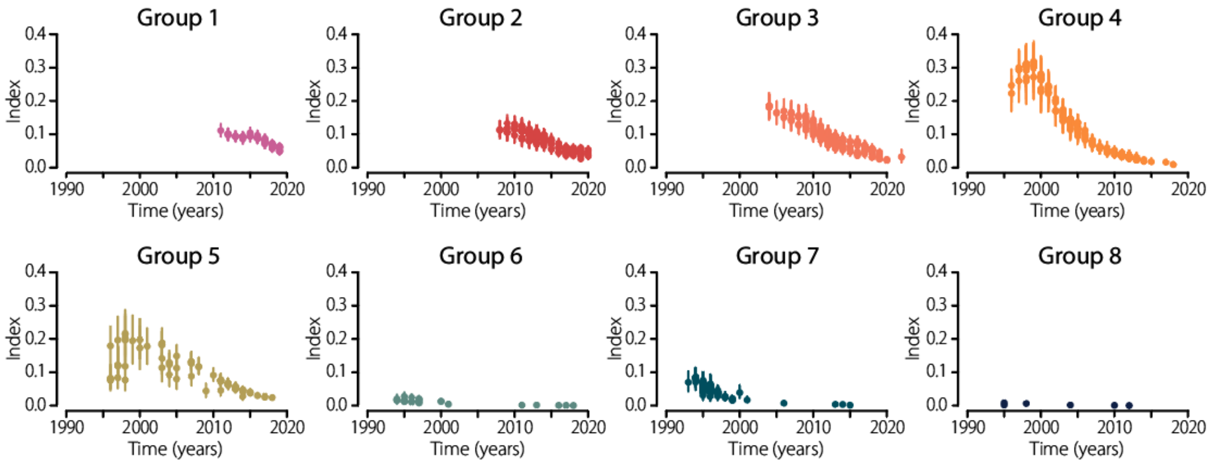

Supplementary Figure 15: Sensitivity analysis: *B. pertussis Index* dynamics over the posterior density of trees

We present the *Index* dynamics computed over 3000 trees from the BEAST posterior of *B. pertussis*. Dots and bars denote the median and 95% credible interval of the *Index* values. We plot only the *Index* of tips as we can summarise their values over the whole posterior.

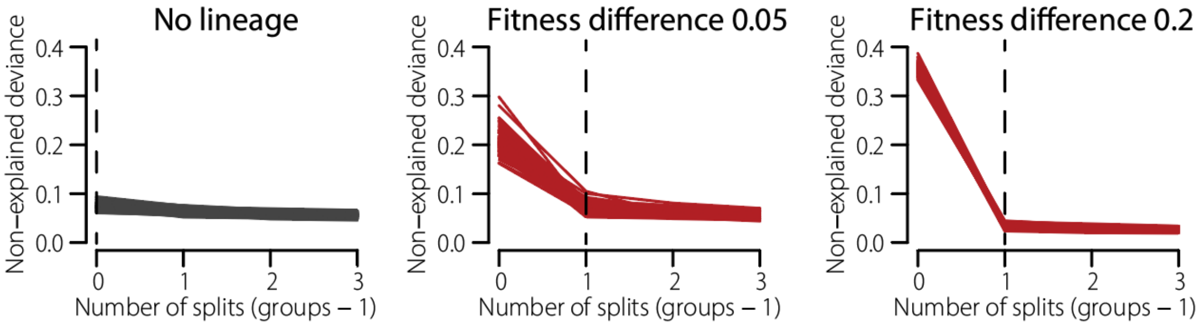

Supplementary Figure 16: Simulation study: non-explained deviance as a function of the number of groups in the lineage detection algorithm.

We simulated trees with or without an emerging lineage - from left to right: no lineage, fitness difference of 0.05/time unit, and fitness difference of 0.2/time unit. We present here the proportion of non-explained deviance by the models with different numbers of groups. Each simulation is plotted as a line (100 lines per scenario). The colour of the line indicates if a lineage was detected (grey: no lineage, red: one lineage detected). Lineages are detected if the non-explained deviance continues to decrease. Dashed lines denote the number of groups chosen.

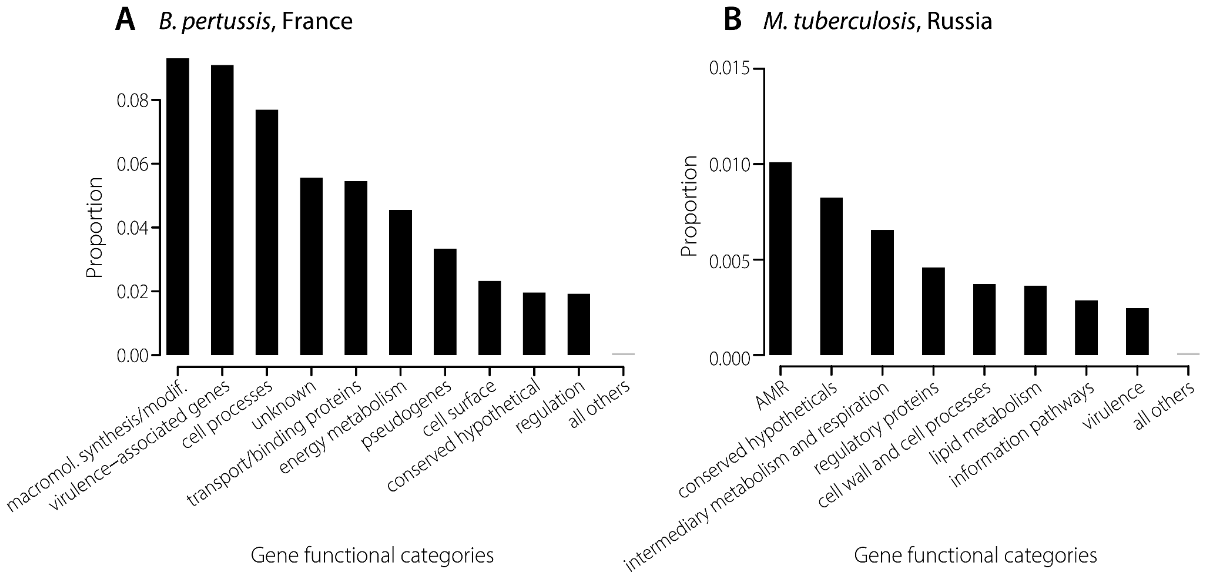

Supplementary Figure 17: Proportion of synonymous mutations that are lineage-defining, by gene functional categories, for *B. pertussis* and *M. tuberculosis*

Similarly to Figure 4K-L, we plot the proportion of synonymous mutations that are lineage-defining within each functional category, for (A) *B. pertussis* and (B) *M. tuberculosis* [35,36]. For *M. tuberculosis*, we only considered the most recent lineages 1 and 2. As expected, we find no statistical differences, as opposed to Figure 4K-L.

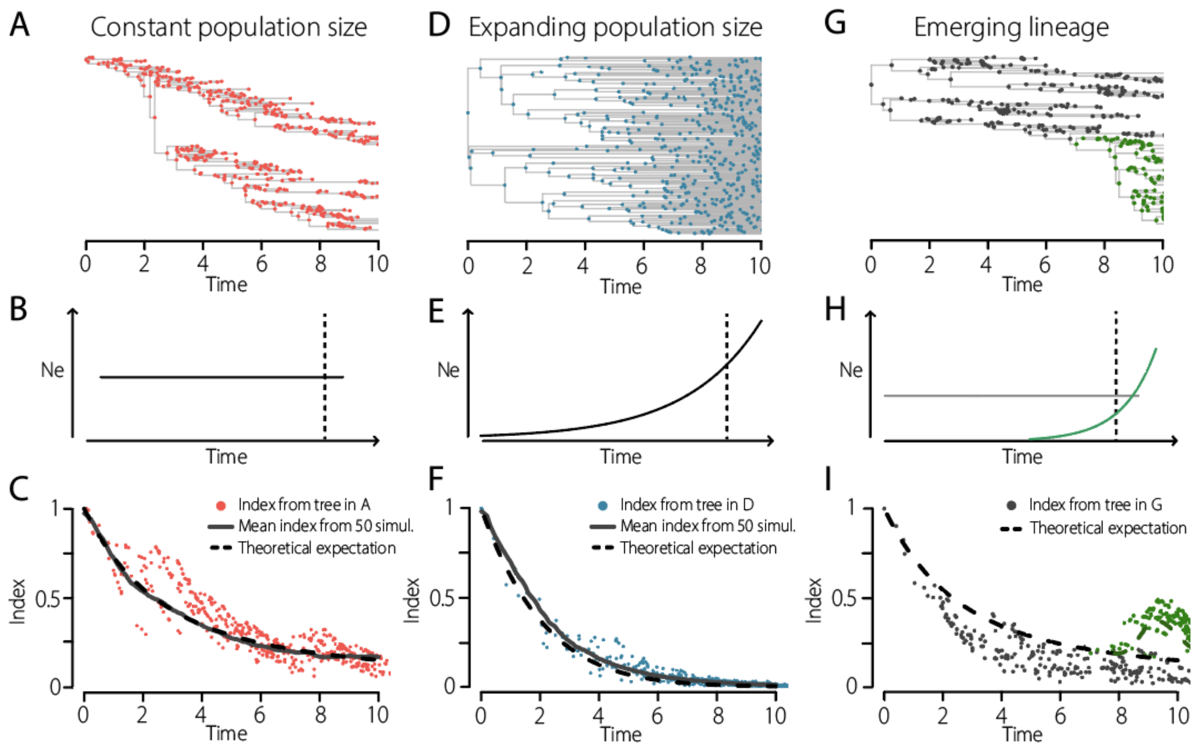

Supplementary Figure 18: Illustration of the index behaviour in different population histories. Similarly to Figure S21, we illustrate here the behaviour of the index. In each case, we simulate trees and compute the index on them. **(A-C)** Constant population size. Simulated time-resolved tree, under a birth-death model with equal probability of birth and death, i.e, constant population size on average. (B) Effective population size used in the simulation. (C) Index through time. **(D-F)** Exponential population size. **(G-I)** Case of an emerging, exponentially growing, lineage in a population of constant effective size. Colours denote each simulation. Dashed lines: expected dynamics given equations in the Methods. Solid lines: mean over the 50 simulations.

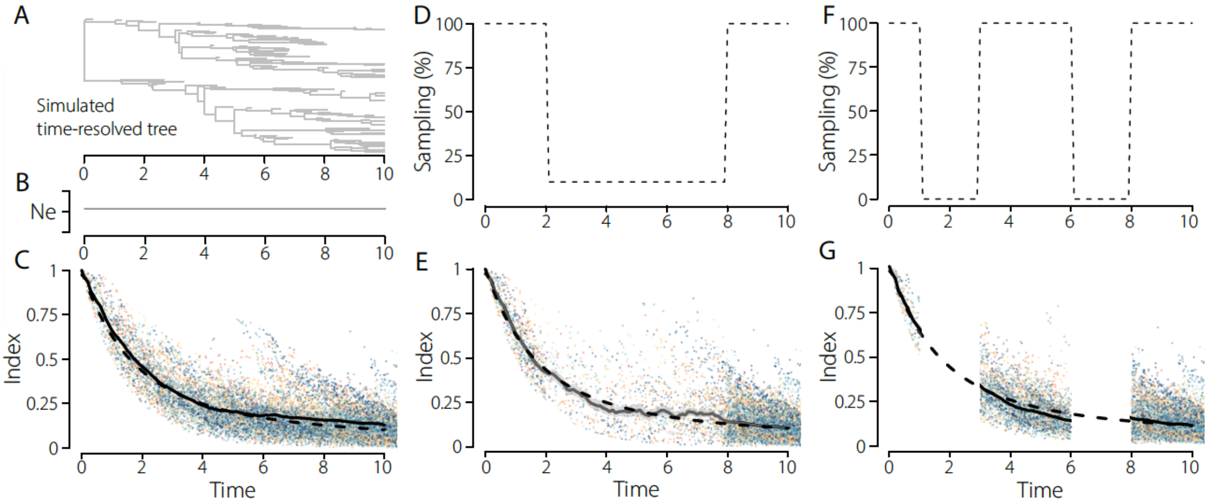

Supplementary Figure 19: Robustness to sampling schemes, from simulation study.

We assess the robustness of the index computation to sampling intensity. **(A-C)** Simulations with no sampling bias. 50 simulations were performed. The tree in A represents one simulation. B represents the effective population size trend: constant. **(D-E)** For each simulation, only 10% of the sequences from year 2-8 were used to compute the index. **(F-G)** No sequences from years 1-3 or 6-8 were used to compute the index. In C, E and G, colours denote each simulation. Dashed lines: expected dynamics given equations in the Methods. Solid lines: mean over the 50 simulations, for the different sampling biases.

Supplementary Tables

Pathogen	SARS-CoV-2	Influenza H3N2	*B. pertussis*	*M. tuberculosis*
Genome length	29903	1701	4086189	4411532
Substitution rate (substitution per site per year)	$8.1\ 10^{-4}$	$3.82\ 10^{-3}$	$2.5\ 10^{-7}$	$4.6\ 10^{-8}$
Time scale (years)	0.15	0.4	2	30
Kernel bandwidth *b*	0.91	0.87	0.84	0.94

Supplementary Table 1: Genome lengths, substitution rates, timescales and bandwidths used in this study.

We present the list of the different parameters that we use to compute the index. The function "*index.bandwidth()*" in the GitHub library allows to compute the kernel bandwidth given a genome length, a substitution rate and a timescale. The kernel bandwidth is dimensionless.

VOCs			Mu	Lambda	Iota	Eta	Epsilon	Kappa	EU1	Beta	Alpha	Gamma	Delta	Omicron								
**Nextclade**			**21H**	**21G**	**21F**	**21D**	**21C**	**21B**	**20E**	**20H**	**20I**	**20J**	**21A-I-J**	**21K**	**22C**	**22A**	**21L**	**22B**	**22E**	**22D**	**22F**	**23A**
**Pango**		**wt**	**B.1.621**	**C.37**	**B.1.526**	**B.1.525**	**B.1.427**	**B.1.617.1**	**B.1.177**	**B.1.351**	**B.1.1.7**	**P.1**	**B.1.617.2**	**BA.1**	**BA.2.12.1**	**BA.4**	**~BA.2**	**BA.5**	**BQ.1**	**BA.2.75**	**XBB**	**XBB.1.5**
**Automatic clades**	**1**	0	0	0	0	0	0	0	0	0	0	0	0	0	0	0	0	0	0	0	18	149
	**2**	0	0	0	0	0	0	0	0	0	0	0	0	0	0	0	0	0	0	0	38	0
	**3**	0	0	0	0	0	0	0	0	0	0	0	0	0	0	0	0	0	0	115	0	0
	**4**	0	0	0	0	0	0	0	0	0	0	0	0	0	0	0	0	6	243	0	0	0
	**5**	0	0	0	0	0	0	0	0	0	0	0	0	0	0	0	0	764	0	0	0	0
	**6**	0	0	0	0	0	0	0	0	0	0	0	0	0	63	103	600	0	0	0	0	0
	**7**	3	0	0	0	0	0	0	0	0	0	0	0	471	0	0	0	0	0	0	0	0
	**8**	0	0	0	0	0	0	0	0	0	0	0	172	0	0	0	0	0	0	0	0	0
	**9**	0	0	0	0	0	0	0	0	0	0	0	889	0	0	0	0	0	0	0	0	0
	**10**	0	0	0	0	0	0	0	0	0	0	91	0	0	0	0	0	0	0	0	0	0
	**11**	0	0	0	0	0	0	0	0	0	339	0	0	0	0	0	0	0	0	0	0	0
	**12**	0	0	0	0	0	0	0	0	91	0	0	0	0	0	0	0	0	0	0	0	0
	**13**	1661	27	13	5	35	11	5	57	0	0	0	0	0	0	0	0	0	0	0	0	0

	**14**	288	0	0	0	0	0	0	0	0	0	0	0	0	0	0	0	0	0	0	0	0

Supplementary Table 2: SARS-CoV-2 contingency table comparing the automatic lineages to those previously identified.
Numbers indicate the counts of tips and internal nodes belonging to each category.

Global clade		wt	3C	3C.2a 4	3C.2	3C.3	3C.3b	3C.3a	3C.2a	3C.2a 2	3C.2a 1a	3C.2a 1	3C.2a 3	3C.2a 1b.1	3C.2a 1b.1a	3C.3a 1	3C.2a 1b.2a	2d	2c	1	3C.2a 1b.2	3C.2a 1b.2b	2	3C.2a 1b.1b	2b	2a
**Automatic clades**	**1**	0	0	0	0	0	0	0	0	0	0	0	0	0	0	0	0	0	0	0	0	0	19	0	51	311
	**2**	0	0	0	0	0	0	0	0	0	0	0	0	0	0	0	0	0	0	0	0	0	0	99	0	0
	**3**	0	0	0	0	0	0	0	0	0	0	0	0	0	0	0	0	0	0	0	43	63	0	0	0	0
	**4**	0	0	0	0	0	0	0	0	0	0	0	0	0	0	39	0	0	0	0	0	0	0	0	0	0
	**5**	0	0	0	0	0	0	0	0	0	0	0	0	0	0	0	39	11	21	67	28	0	14	0	0	0
	**6**	0	0	0	0	0	0	0	0	0	0	0	0	139	55	0	0	0	0	0	0	0	0	0	0	0
	**7**	0	0	0	0	0	0	0	0	0	0	60	0	83	0	0	0	0	0	0	0	0	0	0	0	0
	**8**	0	0	0	0	0	0	0	0	0	0	0	83	0	0	0	0	0	0	0	0	0	0	0	0	0
	**9**	0	0	0	0	0	0	0	94	0	31	79	0	0	0	0	0	0	0	0	0	0	0	0	0	0
	**10**	0	0	0	0	0	0	0	59	189	0	0	0	0	0	0	0	0	0	0	0	0	0	0	0	0
	**11**	0	0	0	0	0	0	0	257	0	0	0	2	0	0	0	0	0	0	0	0	0	0	0	0	0
	**12**	0	0	0	0	0	0	124	0	0	0	0	0	0	0	16	0	0	0	0	0	0	0	0	0	0
	**13**	0	0	0	0	261	27	6	0	0	0	0	0	0	0	0	0	0	0	0	0	0	0	0	0	0
	**14**	0	57	25	76	36	0	0	27	0	0	0	0	0	0	0	0	0	0	0	0	0	0	0	0	0
	**15**	87	0	0	0	0	0	0	0	0	0	0	0	0	0	0	0	0	0	0	0	0	0	0	0	0
	**16**	53	12	0	0	0	0	0	0	0	0	0	0	0	0	0	0	0	0	0	0	0	0	0	0	0
	**17**	0	96	0	0	0	0	0	0	0	0	0	0	0	0	0	0	0	0	0	0	0	0	0	0	0
	**18**	97	0	0	0	0	0	0	0	0	0	0	0	0	0	0	0	0	0	0	0	0	0	0	0	0
	**19**	9	0	0	0	0	0	0	0	0	0	0	0	0	0	0	0	0	0	0	0	0	0	0	0	0
	**20**	6	0	0	0	0	0	0	0	0	0	0	0	0	0	0	0	0	0	0	0	0	0	0	0	0

Supplementary Table 3: H3N2 contingency table comparing the automatic lineages to those previously identified.

Numbers indicate the counts of tips and internal nodes belonging to each category.

Genotypes		ptxP3/fim3-2	ptxP3/fim3-1	ptxP1/fim3-1
**Automatic clades**	**1**	0	155	0
	**2**	529	0	0
	**3**	0	682	0
	**4**	584	0	0
	**5**	0	197	0
	**6**	0	0	56
	**7**	0	30	207
	**8**	0	0	15

Supplementary Table 4: ***B. pertussis*** **contingency table comparing the automatic lineages to those previously identified.**
Numbers indicate the counts of tips and internal nodes belonging to each category.

Lineages		CAS	EAI	Haarlem	E-A	Ural	LAM	Beijing	Clade A	Clade B
**Automatic clades**	**1**	0	0	0	0	0	0	0	0	515
	**2**	0	0	0	0	0	0	0	227	0
	**3**	0	0	0	0	0	0	513	4	0
	**4**	0	0	0	0	0	143	0	0	0
	**5**	0	0	0	0	131	0	0	0	0
	**6**	0	0	0	37	0	0	0	0	0
	**7**	0	0	0	45	0	0	0	0	0
	**8**	0	0	0	140	0	0	0	0	0
	**9**	0	0	0	12	62	32	0	0	0
	**10**	0	0	81	0	0	0	0	0	0
	**11**	0	0	0	36	0	0	0	0	0
	**12**	6	7	0	2	0	0	0	0	0

Supplementary Table 5: ***M. tuberculosis*** **contingency table comparing the automatic lineages to those previously identified.**
Numbers indicate the counts of tips and internal nodes belonging to each category. LAM denotes the Latin American-Mediterranean lineage, E-A the Euro-American lineage, EAI the East African Indian lineage and CAS the Central Asian Strain lineage.

Supplementary Table 6: SARS-CoV-2 lineage-defining mutations.
For each lineage, the set of defining amino-acid substitutions is given. Mutations are separated in three groups based on their locations in the genome, for convenience: "Spike", "E, N and M"and "ORFs". This table is provided as a csv file.

Supplementary Table 7: H3N2 lineage-defining mutations.
For each lineage, the set of defining amino-acid substitutions in the HA protein is given. Mutations are separated in three groups based on their locations in the protein, for convenience: "Sig", "HA1"and "HA2". This table is provided as a csv file.

Supplementary Table 8: *Bordetella pertussis* lineage-defining mutations in France.
This table lists all the *Bordetella pertussis* lineage-defining non-synonymous substitutions and mutations in the promoter regions. Information on the locus tag, gene functional category, gene product and gene name is included when available. This table is provided as a csv file.

Supplementary Table 9: *Mycobacterium tuberculosis* lineage-defining mutations in Samara, Russia.
This table lists all the *Mycobacterium tuberculosis* lineage-defining non-synonymous substitutions and mutations in the promoter regions. Information on the gene id, gene functional category, gene product and gene name is included when available. This table is provided as a csv file.

Supplementary Table 10: Isolates of SARS-CoV-2
This table lists all the 3129 whole genome SARS-CoV-2 sequences used in this study. For each sequence, we list its identifier, GISAID accession number, collection date, country of isolation and Nextstrain clade. This table is provided as a csv file.

Supplementary Table 11: Isolates of H3N2
This table lists all the 1476 H3N2 Hemagglutinin sequences used in this study. For each sequence, we list its identifier, GISAID accession number, collection date, location of isolation and global clade. This table is provided as a csv file.

Supplementary Table 12: Isolates of *Bordetella pertussis*
This table lists all the 1248 whole genome *B. pertussis* sequences from France[3,38–41] and the Tohama I reference genome. For each sequence, we list its identifier, accession number, collection year, country of isolation and genotype. This table is provided as a csv file.

Supplementary Table 13: Isolates of *Mycobacterium tuberculosis*
This table lists all the 997 whole genome *M. tuberculosis* sequences from Samara, Russia[20] and the H37Rv reference genome. For each sequence, we list its accession number, collection year, country of isolation and clade. This table is provided as a csv file.
