# Supplementary material for: Learning the fitness dynamics of pathogens from phylogenies
Source: Nature. 2025 Jan 1;637(8046):683–90. doi: 10.1038/s41586-024-08309-9 (PMC11735385; doi:10.1038/s41586-024-08309-9)
Supplement: Supplementary file 2 — Reporting Summary [file 41586_2024_8309_MOESM2_ESM.pdf]

nature portfolio

Corresponding author(s): Noemie Lefrancq

Last updated by author(s): Oct 18, 2024

Reporting Summary

Nature Portfolio wishes to improve the reproducibility of the work that we publish. This form provides structure for consistency and transparency in reporting. For further information on Nature Portfolio policies, see our Editorial Policies and the Editorial Policy Checklist.

Statistics

For all statistical analyses, confirm that the following items are present in the figure legend, table legend, main text, or Methods section.

n/a	Confirmed	
☐	☒	The exact sample size (n) for each experimental group/condition, given as a discrete number and unit of measurement
☐	☒	A statement on whether measurements were taken from distinct samples or whether the same sample was measured repeatedly
☐	☒	The statistical test(s) used AND whether they are one- or two-sided<br>*Only common tests should be described solely by name; describe more complex techniques in the Methods section.*
☐	☒	A description of all covariates tested
☐	☒	A description of any assumptions or corrections, such as tests of normality and adjustment for multiple comparisons
☐	☒	A full description of the statistical parameters including central tendency (e.g. means) or other basic estimates (e.g. regression coefficient) AND variation (e.g. standard deviation) or associated estimates of uncertainty (e.g. confidence intervals)
☒	☐	For null hypothesis testing, the test statistic (e.g. F, t, r) with confidence intervals, effect sizes, degrees of freedom and P value noted<br>*Give P values as exact values whenever suitable.*
☐	☒	For Bayesian analysis, information on the choice of priors and Markov chain Monte Carlo settings
☒	☐	For hierarchical and complex designs, identification of the appropriate level for tests and full reporting of outcomes
☒	☐	Estimates of effect sizes (e.g. Cohen's d, Pearson's r), indicating how they were calculated

Our web collection on statistics for biologists contains articles on many of the points above.

Software and code

Policy information about availability of computer code

Data collection	For each pathogen, we compiled a dataset to investigate the changes in the population composition. For SARS-CoV-2 and Influenza H3N2, we extracted the datasets from the publicly available NextStrain37 timed-resolved phylogenies accessed on 14 April 2023. These datasets are sub-samples from all publicly available sequences in GISAID, to represent the diversity as much as possible (we used the 'all-time' dataset for SARS-CoV-2 and the '12y' one for H3N2). In all, we have 3129 whole genome SARS-CoV-2 sequences sampled from 26 December 2019 to 3 April 2023, and 1476 Influenza H3N2 Hemagglutinin (HA) sequences from 1 January 2005 to 3 April 2023 (Supplementary Tables 10-11). For B. pertussis, we used 1248 sequences from 1953 to 2022, collected by the National Reference Center (NRC) for Whooping Cough and Other Bordetella Infections in France (Supplementary Table 12). This dataset is composed of 1023 sequences previously published3,38–41 and 225 newly sequenced isolates. The new isolates have been sequenced with the same methods as previously described3. This dataset is representative of the B. pertussis diversity in France as the NRC is receiving isolates from 42 sentinelle hospitals throughout France. For M. tuberculosis, we used 997 previously published sequences, isolated in 2008-2010 in Samara, Russia20. This dataset is also representative of M. tuberculosis sequence diversity at that location as isolates were prospectively collected from individual patients living in the region and representative of the entire population (Supplementary Table 13).
Data analysis	To create the multi-sequence alignment for each pathogen, the following softwares and algorithms were used: MAFFT (v7.309), Cutadapt (v3.4), FastQC (v0.11.9), BWA-MEM (v0.7.17), GATK (v4.2.0.0), PHASTER, Gubbins (v3.3.0).<br>To reconstruct the time-resolved phylogenetic trees, the following softwares and algorithms were used: IQ-tree (v2.1.0), BEAST (v1.10.4), BEAGLE library (v4.0.0), Tracer (v1.7.2), BactDating (v1.1).<br>For the main code analysis, we wrote the code in R v4.1.2, and used the package mgcv v1.8 to build the detection algorithm.<br>We compared phylowave to fastbaps v1.0.8 and treestructure.

Code availability statement:
Code to replicate the main analyses of this paper is publicly available at https://zenodo.org/records/13952222 [Ref: 62]. General guidelines to use phylowave and a step-by-step example are included in Supplementary Text 3-4.

For manuscripts utilizing custom algorithms or software that are central to the research but not yet described in published literature, software must be made available to editors and reviewers. We strongly encourage code deposition in a community repository (e.g. GitHub). See the Nature Portfolio guidelines for submitting code & software for further information.

Data

Policy information about availability of data

All manuscripts must include a data availability statement. This statement should provide the following information, where applicable:

- Accession codes, unique identifiers, or web links for publicly available datasets
- A description of any restrictions on data availability
- For clinical datasets or third party data, please ensure that the statement adheres to our policy

Data availability statement:
All B. pertussis sequences generated for this study were deposited in ENA, with accession numbers and metadata attached for each individual sequence available in Supplementary Table 10. All sequences and metadata used in this study, including the reference sequences, are listed in Supplementary Tables 10-13. All sequences are publicly available online on GenBank and ENA (B. pertussis, M. tuberculosis20) or GISAID (H3N2, SARS-CoV-2). The Supplementary Tables 10-13 are also available online in the repository: https://zenodo.org/records/13952222 [Ref: 62].

Research involving human participants, their data, or biological material

Policy information about studies with human participants or human data. See also policy information about sex, gender (identity/presentation), and sexual orientation and race, ethnicity and racism.

Reporting on sex and gender	NA
Reporting on race, ethnicity, or other socially relevant groupings	NA
Population characteristics	NA
Recruitment	NA
Ethics oversight	Data on SARS-CoV-2, H3N2 and M. tuberculosis were previously published. B. pertussis data was obtained by the French National Reference Centre for Whooping Cough and Other Bordetella infections, which is authorised to conduct sample collection.

Note that full information on the approval of the study protocol must also be provided in the manuscript.

Field-specific reporting

Please select the one below that is the best fit for your research. If you are not sure, read the appropriate sections before making your selection.

☐ Life sciences ☐ Behavioural & social sciences ☒ Ecological, evolutionary & environmental sciences

For a reference copy of the document with all sections, see nature.com/documents/nr-reporting-summary-flat.pdf

Ecological, evolutionary & environmental sciences study design

All studies must disclose on these points even when the disclosure is negative.

Study description	For each pathogen, we compiled a dataset to investigate the changes in population composition with our novel framework.
Research sample	Whole-genomes of SARS-CoV-2, Bordetella pertussis and Mycobacterium tuberculosis, and HA gene of H3N2.
Sampling strategy	For each pathogen studied, we gathered a dataset that is representative of that pathogen's genetic diversity.
Data collection	Data on SARS-CoV-2, H3N2 and M. tuberculosis were previously published.B. pertussis data was obtained by the French National Reference Centre for Whooping Cough and Other Bordetella infections.
Timing and spatial scale	SARS-CoV-2: 2019-2023, worldwide,<br>H3N2: 2005-2023, worldwide,<br>B. pertussis: 1953-2022, France<br>M. tuberculosis: 2008-2010, Russia

Data exclusions	NA
Reproducibility	We only used natural experiment in this study (pathogen circulating in human population) - analysis is reproducible using codes
Randomization	We only used natural experiment in this study (pathogen circulating in human population)
Blinding	We only used natural experiment in this study (pathogen circulating in human population)

Did the study involve field work? ☐ Yes ☒ No

Reporting for specific materials, systems and methods

We require information from authors about some types of materials, experimental systems and methods used in many studies. Here, indicate whether each material, system or method listed is relevant to your study. If you are not sure if a list item applies to your research, read the appropriate section before selecting a response.

Materials & experimental systems

n/a	Involved in the study
☒	☐ Antibodies
☒	☐ Eukaryotic cell lines
☒	☐ Palaeontology and archaeology
☒	☐ Animals and other organisms
☒	☐ Clinical data
☒	☐ Dual use research of concern
☒	☐ Plants

Methods

n/a	Involved in the study
☒	☐ ChIP-seq
☒	☐ Flow cytometry
☒	☐ MRI-based neuroimaging

Plants

Seed stocks	NA
Novel plant genotypes	NA
Authentication	NA
